# Mechanical and Microstructural Performance of Hydraulic Lime-Based Mortars Incorporating Mechanically Milled Mineral Additives and Graphene Nanoplatelets

**DOI:** 10.3390/ma19214713

**Published:** 2026-11-05

**Authors:** Ali Ikbal Tutar, Ferit Cakir, Tuncay Simsek, Hasan İlhan

**Affiliations:** 1 Department of Civil Engineering, Gebze Technical University, 41400 Kocaeli, Türkiye; 2 Motor Vehicles and Transport Technologies Department, Kirikkale University, 71450 Kirikkale, Türkiye; 3 Biotechnology Institute, Biotechnology Department, Ankara University, 06135 Ankara, Türkiye

**Keywords:** hydraulic lime-based mortars, mechanical milling, graphene nanoplatelets, planetary ball milling, mechanical performance, microstructural characterization, historic masonry conservation

## Abstract

**Highlights:**

- Mechanically milled mineral additives and graphene nanoplatelets were investigated in hydraulic lime-based mortars.
- IM-3% achieved the highest compressive strength, with a 4.4% increase over the reference mortar.
- SCL-1% improved flexural strength by 28.0%, while GN-1% achieved a 19.1% increase.
- Mechanical performance exhibited a strong dependence on additive type and dosage.
- SEM-EDS, XRD, and FT-IR revealed variations in matrix morphology and compositional characteristics.

**Abstract:**

The development of advanced hydraulic lime-based mortars incorporating mineral additives and graphene nanoplatelets offers potential opportunities for improving material performance in the conservation of historic masonry structures. This study investigates the mechanical and microstructural performance of hydraulic lime-based mortars incorporating mechanically milled mineral additives and commercially available graphene nanoplatelets (GNPs). Hydraulic lime, brick powder, repair mortar, injection mortar, slaked construction lime, and gypsum plaster were processed using planetary ball milling under identical conditions, whereas graphene nanoplatelets were incorporated without additional mechanical treatment. The additives were introduced at 1%, 2%, and 3% by mass of the original hydraulic lime binder. Compressive and flexural strengths were evaluated after 28 days of curing, complemented by scanning electron microscopy and energy-dispersive spectroscopy (SEM-EDS), X-ray diffraction (XRD), and Fourier-transform infrared spectroscopy (FT-IR). The results demonstrated that mechanical performance was strongly influenced by additive type and dosage. The mortar containing 3% injection mortar additive (IM-3%) achieved the highest compressive strength of 9.75 MPa, representing a 4.4% increase over the reference mixture, together with a 25.8% improvement in flexural strength. The incorporation of 1% slaked construction lime (SCL-1%) produced the highest mean flexural strength of 3.02 MPa, corresponding to a 28.0% increase, whereas 1% graphene nanoplatelets (GN-1%) enhanced flexural strength by 19.1% despite reducing compressive strength. Most other modified mixtures exhibited lower mechanical strengths than the reference mortar. Microstructural characterization revealed variations in matrix morphology and local elemental distribution, while the principal carbonate- and silicate-related characteristics were largely retained. These findings demonstrate the composition-dependent mechanical response of hydraulic lime-based mortars incorporating mechanically milled mineral additives and graphene nanoplatelets, providing a basis for further investigations into their long-term performance and compatibility with historic masonry materials.

## 1. Introduction

Historic masonry structures constitute an essential part of the world’s architectural and cultural heritage, reflecting the construction techniques, material characteristics, and engineering knowledge of different historical periods. Over time, these structures are subjected to various deterioration mechanisms arising from environmental exposure, moisture fluctuations, temperature variations, material aging, and static and dynamic loading. Such influences may progressively reduce the mechanical integrity of masonry components, promote crack development, and compromise the overall structural stability of historic buildings [1]. Consequently, the conservation and structural rehabilitation of historic masonry require intervention strategies that ensure adequate mechanical performance while preserving the physical, chemical, and mechanical compatibility of repair materials with the original construction fabric.

One of the principal challenges in the conservation of historic masonry is the presence of internal discontinuities, particularly in multi-leaf stone masonry walls. These structures commonly consist of two external masonry leaves surrounding an irregular inner core composed of rubble, fragmented mortar, and poorly consolidated materials. The presence of internal voids, insufficient transverse connections, and degraded bonding conditions may prevent the individual leaves from developing an effective composite structural response. Consequently, such walls can exhibit reduced shear resistance, limited out-of-plane stability, and increased vulnerability under seismic loading. Previous investigations have highlighted the potential of grout injection and other consolidation techniques to improve the structural integrity of multi-leaf masonry, while also demonstrating that their effectiveness depends on the characteristics of the existing masonry and the properties of the injected materials [2,3,4]. Among the available conservation techniques, lime-based grout injection has received considerable attention because of its potential to restore internal continuity while maintaining compatibility with historic masonry materials.

Hydraulic lime-based mortars have been widely investigated as repair and consolidation materials because of their hydraulic setting characteristics, potential vapor permeability, and compatibility with traditional mineral-based construction materials. Compared with Portland cement-rich formulations, appropriately designed hydraulic lime mortars may provide a more favorable balance between strength development, deformability, and moisture transport characteristics. However, their performance is strongly dependent on binder composition, aggregate characteristics, water content, curing conditions, and the incorporation of supplementary constituents. This sensitivity to mixture composition was also observed by [5], who examined how binder and aggregate selection and their relative proportions affect the mechanical properties of lime-based restoration mortars. For conservation applications, mechanical strength alone cannot determine material suitability, as excessive stiffness, unfavorable moisture transport characteristics, or incompatible chemical compositions may generate additional deterioration mechanisms. Accordingly, the development of hydraulic lime-based mortars requires a comprehensive understanding of the relationships between material composition, mechanical behavior, and microstructural characteristics [6,7,8].

The modification of lime-based binders through mineral additions has emerged as an important strategy for adjusting their physical and mechanical properties. Pozzolanic materials and other supplementary mineral constituents may influence hydration processes, particle packing, pore structure, and the development of binding phases. Previous studies have investigated the incorporation of silica fume, fly ash, and other mineral additions into hydraulic lime-based grouts, demonstrating that their effectiveness depends on the chemical and physical characteristics of the additives, their proportions, and the composition of the reference binder [9,10,11,12]. These investigations indicate that mineral additions do not necessarily produce uniform improvements in mechanical performance. Instead, the resulting behavior reflects complex interactions among the constituent materials, highlighting the importance of systematically comparing different additive types under consistent experimental conditions.

In parallel with conventional mineral additives, considerable attention has been directed toward the incorporation of nanoscale materials into cementitious and lime-based matrices. Nanoparticles may influence material behavior through their high specific surface area, surface interactions, and potential effects on hydration and microstructural development. Depending on their chemical composition and dispersion characteristics, these materials may contribute to changes in pore structure, the distribution of hydration products, and the mechanical response of hardened binders. However, their effects are not universally beneficial and depend strongly on dosage, compatibility with the binder, and the tendency of particles to form agglomerates. Reference [13] investigated the incorporation of nano-Ca(OH)_2_ and nano-SiO_2_ into air lime mortars containing ceramic additions and demonstrated that nanoparticle additions could modify microstructural development and mechanical behavior. Their findings highlighted the complex relationship between nanoparticle incorporation, pozzolanic reactions, carbonation processes, and deformation characteristics. In natural hydraulic lime systems, ref. [14] demonstrated that nano-SiO_2_ could influence early hydration kinetics and subsequently promote changes associated with pozzolanic reactions and strength development. Collectively, these investigations emphasize that the effectiveness of fine and nanoscale additions depends on the characteristics of the host binder and the physicochemical interactions occurring during hardening.

More recently, nano-metakaolin has attracted attention as a potential modifier for natural hydraulic lime-based materials. Investigations by [15,16,17] reported improvements in selected mechanical and microstructural properties of hydraulic lime systems incorporating nano-metakaolin. The reported improvements were associated with the combined effects of fine-particle filling, pozzolanic activity, and changes in hydration product development. Other investigations have examined the influence of nano-TiO_2_ and nano-alumina on lime-based binders. Reference [18] investigated the physicochemical and mechanical behavior of hydraulic lime-metakaolin mortars containing nano-TiO_2_, while ref. [19] demonstrated that the effectiveness of nano-silica and nano-alumina depended on the chemical characteristics of the lime–pozzolan matrix. These studies collectively suggest that the incorporation of finely divided materials can modify binder performance, although the resulting effects cannot be predicted solely from particle size or additive dosage.

Beyond the direct incorporation of manufactured nanoparticles, mechanical milling provides an alternative route for processing conventional mineral materials before their introduction into mortar systems. Planetary ball milling subjects particles to repeated impact, compression, and shear actions, which may alter their morphology, particle size distribution, structural organization, and surface characteristics. Depending on the starting material and processing conditions, mechanical treatment can also induce structural disorder and changes in the reactivity of mineral constituents. Reference [20] investigated the incorporation of ball-milled quarry waste into air lime mortars, demonstrating the potential of mechanically processed mineral constituents to influence material behavior. Reference [21] examined the mechanochemical activation of natural clays and reported changes associated with their pozzolanic activity, while ref. [22] investigated particle size distributions and structural changes in limestone subjected to planetary ball milling. These investigations establish mechanical milling as a relevant processing technique for modifying the characteristics of mineral powders, although the magnitude and nature of the resulting changes remain dependent on material composition and processing parameters.

In addition to mineral-based modifications, graphene nanoplatelets have emerged as a promising class of carbon-based additives for cementitious composite materials. Their layered morphology, high aspect ratio, and intrinsic mechanical properties provide a potential basis for influencing microstructural organization and crack development within hardened matrices. However, the effectiveness of graphene nanoplatelets in mineral binders depends considerably on their dispersion, concentration, interactions with hydration products, and tendency to agglomerate. Inadequate dispersion or excessive addition may introduce heterogeneity and localized defects, potentially offsetting the expected mechanical benefits. These considerations are particularly relevant to hydraulic lime-based mortars, where the balance between strength, deformability, and compatibility must be carefully maintained. Therefore, evaluating graphene nanoplatelets alongside mineral additives under comparable mixture preparation and testing conditions may provide useful insights into their relative mechanical effects.

The use of graphene nanoplatelets in lime-based mortars has also been examined experimentally. Reference [23] investigated their influence on the mechanical and physical properties of calcium lime- and hydraulic lime-based mortars, reporting improvements in selected properties at relatively low addition levels. Their findings suggest that graphene nanoplatelets can modify the response of lime-based materials, although the extent of improvement depends on the formulation and incorporation level. More recently, ref. [24] examined the dispersion of modified graphene in cement-based composites, highlighting the importance of achieving a suitable distribution within the binder matrix. While the two studies concern different material systems, they indicate that the effects of graphene additions cannot be considered independently of mixture composition and dispersion conditions.

Despite the growing body of research concerning mineral additions, nanomaterials, and mechanically processed powders, several aspects of their application in hydraulic lime-based mortars remain insufficiently clarified. Existing studies have primarily investigated selected additives individually or focused on particular processing methods and binder compositions. Comparatively fewer investigations have examined a broad range of mechanically milled mineral constituents alongside graphene nanoplatelets within a common hydraulic lime-based mortar formulation. In particular, the comparative effects of additive type and dosage on compressive strength, flexural strength, and hardened matrix characteristics require further investigation. Moreover, the relationship between mechanical performance and microstructural observations remains difficult to establish because local morphological and compositional measurements do not necessarily represent the behavior of the entire mortar matrix.

To address these considerations, this study investigates the mechanical and microstructural performance of hydraulic lime-based mortars incorporating six mechanically milled mineral additives and commercially available graphene nanoplatelets. The mineral additives were processed under identical planetary ball milling conditions, while graphene nanoplatelets were incorporated as received. Additive contents of 1%, 2%, and 3%, relative to the initial hydraulic lime mass, were investigated through compressive and flexural strength testing at 28 days, supported by SEM-EDS, XRD, and FT-IR characterization of the hardened mortars. The primary objective was to compare the responses associated with different additive types and dosages under a consistent experimental protocol. Since untreated counterparts of the milled additives were not included, the study does not aim to isolate the independent contribution of mechanical milling to mortar performance.

The principal contribution of this study lies in the comparative assessment of different mechanically milled mineral additives and graphene nanoplatelets within a consistent experimental framework, allowing their composition- and dosage-dependent effects on hydraulic lime-based mortar performance to be examined. Rather than assuming that mechanical treatment or additive incorporation necessarily enhances strength, the investigation considers both beneficial and adverse responses and explores their possible relationships with the observed characteristics of the hardened matrix. The findings are expected to contribute to the development of modified hydraulic lime-based materials and provide a basis for subsequent investigations addressing long-term durability, material compatibility, and practical suitability for historic masonry conservation.

## 2. Materials and Methods

### 2.1. Mechanical Milling of Mineral Additives

**Figure 1:**
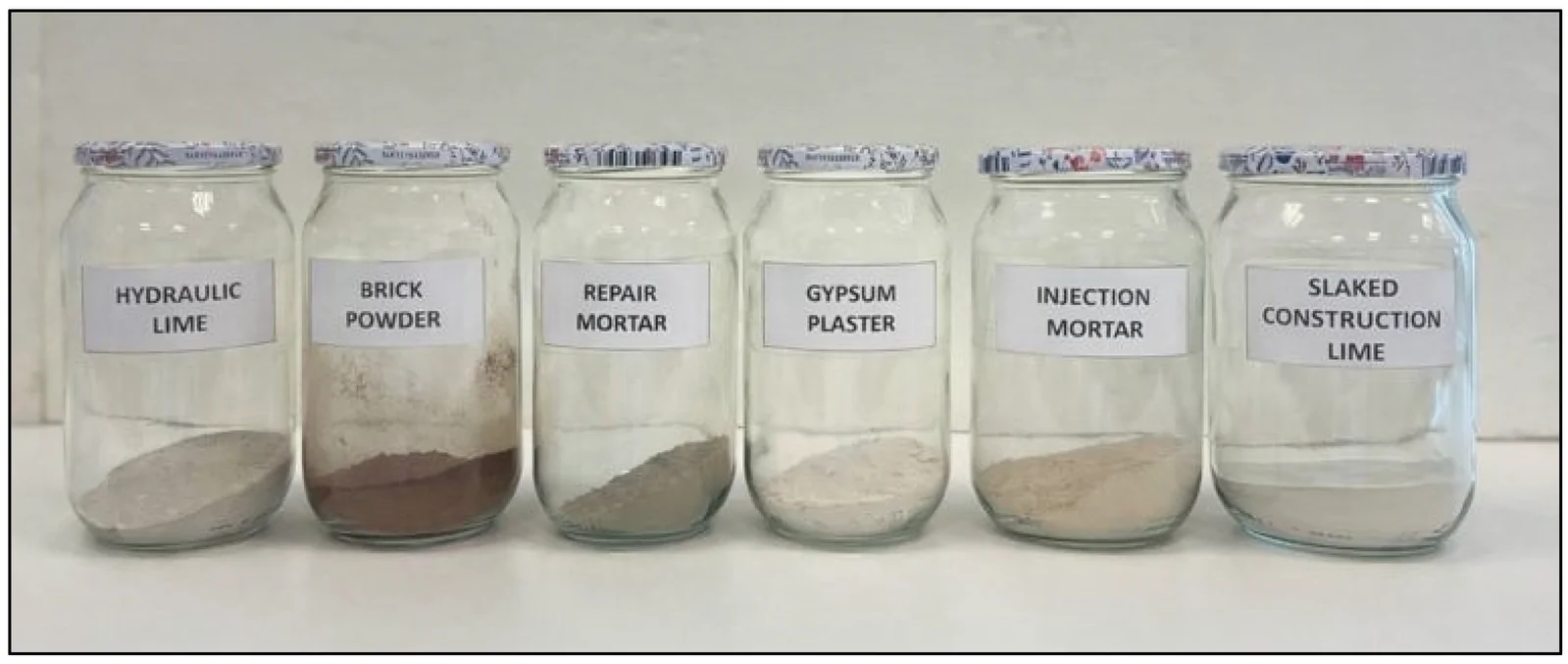
Mechanostructurally treated mineral additives.

**Figure 2:**
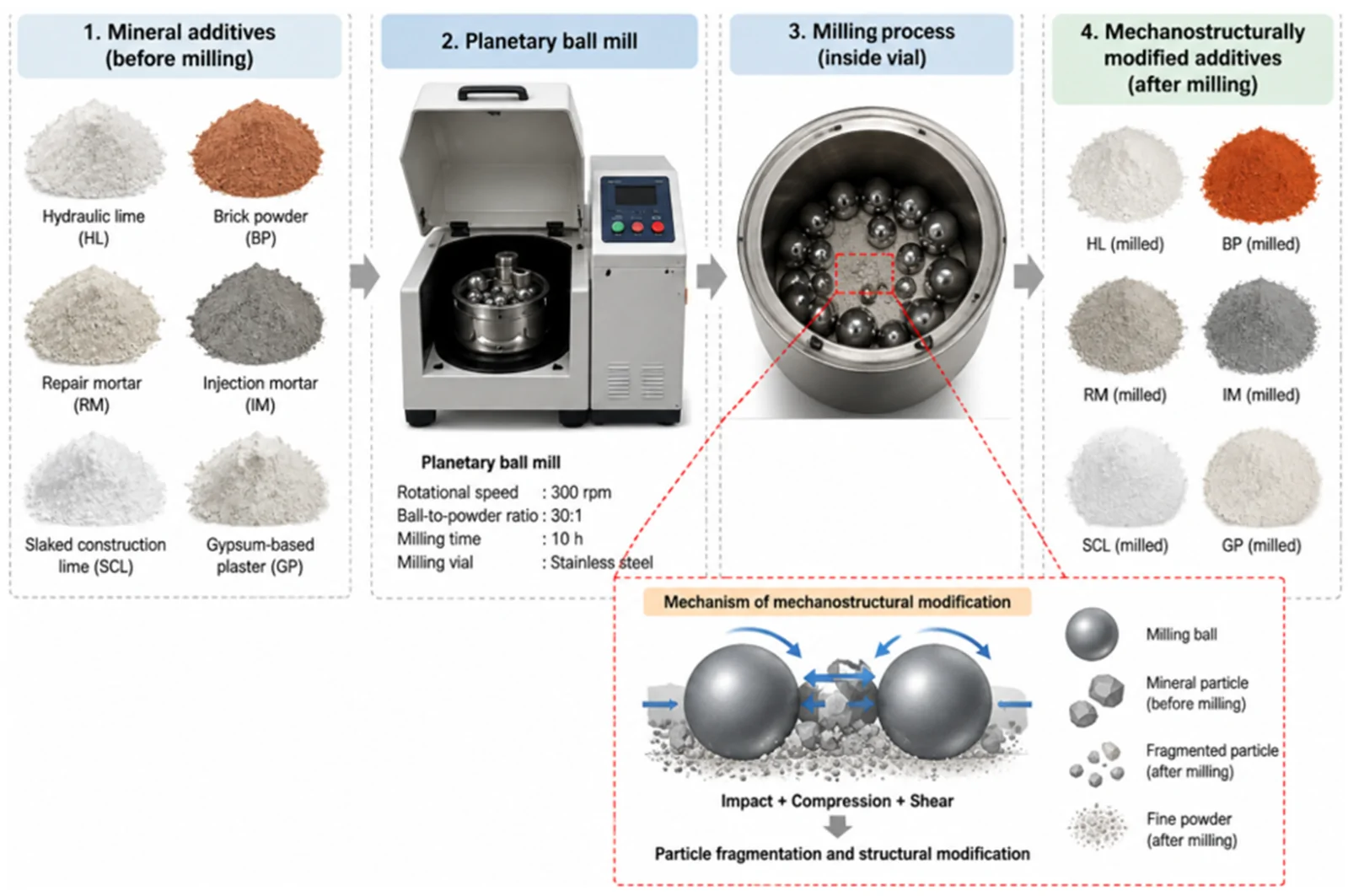
Schematic representation of the planetary ball milling procedure applied to the mineral additives (the particle representations are schematic and do not indicate experimentally measured changes in particle size or morphology).

The mineral additives investigated in this study included hydraulic lime (HL), brick powder (BP), repair mortar (RM), injection mortar (IM), slaked construction lime (SCL), and gypsum-based plaster (GP). Each commercially available material was processed separately using a laboratory-scale planetary ball mill before incorporation into the mortar mixtures. The investigated materials and the milling procedure are illustrated in Figure 1 and Figure 2, respectively. Selected schematic elements in Figure 2, particularly the conceptual representation of particle interactions and fragmentation during planetary ball milling, were generated with the assistance of artificial intelligence (AI). These elements were used only for illustrative purposes and do not represent experimental observations or measured data.

Mechanical milling was performed in a stainless-steel vial at a rotational speed of 300 rpm, with a ball-to-powder mass ratio of 30:1 and a cumulative milling duration of 10 h. The same processing conditions were applied to all six mineral additives to maintain a consistent preparation procedure.

Planetary ball milling subjects particles to repeated impact, compression, and shear forces, which may influence particle size, morphology, surface characteristics, and structural disorder, depending on the material and processing conditions. In the present study, however, particle-size distributions, specific surface areas, and structural characteristics were not measured before and after milling. Consequently, the extent of milling-induced modification cannot be quantified, and the processing conditions should not be interpreted as evidence of a particular reduction in particle size or increase in reactivity. The term “mechanically milled” is therefore used to describe the applied processing procedure rather than a verified structural transformation. Previous investigations have demonstrated the potential of intensive ball milling to influence the physical and structural characteristics of carbonate- and aluminosilicate-bearing materials [20,21,22].

Following the milling procedure, the processed mineral powders were mechanically blended under dry conditions to reduce particle clustering before their incorporation into the hydraulic lime-based mortar mixtures. Commercial graphene nanoplatelets (GNPs) were obtained from Nanografi Co. (Ankara, Türkiye; product code: NG01GNP0105) and used without further mechanical processing. According to the manufacturer’s specifications, the GNPs had an approximate thickness of 5 nm, an average lateral diameter of 18 µm, a specific surface area of 170 m^2^/g, and a purity of 99.9%. Their reported electrical conductivity ranged from 1100 to 1600 S/m.

### 2.2. Preparation of Mixtures

The reference hydraulic lime-based mortar mixture, designated as PM, was prepared using the proportions presented in Table 1. The reference formulation consisted of 1033 kg of hydraulic lime, 362 kg of water, and 665 kg of porcelain aggregate per cubic metre of mortar. Porcelain spheres with a uniform diameter of 5 mm were used as the sole aggregate component, without the incorporation of natural sand or crushed stone. Here, additive percentages are calculated relative to the mass of hydraulic lime in the reference mortar (PM). Mechanically milled mineral additives and graphene nanoplatelets were incorporated separately, without replacing the original constituents. The hydraulic lime, water, and aggregate contents were maintained constant across all mixtures.

The use of uniformly sized porcelain spheres as the sole aggregate was intended to minimize variations in aggregate size, shape, and surface characteristics across the experimental mixtures. This approach provided a controlled laboratory configuration for comparing the effects of additive type and dosage while maintaining identical aggregate conditions. However, porcelain spheres differ from the naturally graded aggregates typically employed in hydraulic lime-based conservation mortars in terms of particle geometry, surface characteristics, and aggregate packing. These differences may influence the aggregate–matrix interface, mechanical response, and fresh-state behavior. Accordingly, the present results should be interpreted as comparative findings obtained from a specific laboratory formulation rather than as directly transferable performance values for conventional conservation mortars.

The water-to-hydraulic lime mass ratio was maintained at approximately 0.35. The same reference formulation was adopted for all modified mixtures, with the quantities of hydraulic lime, water, and porcelain aggregate remaining constant throughout the experimental program.

The mechanically milled mineral additives, including HL, BP, RM, IM, and SCL, were incorporated at proportions of 1%, 2%, and 3% by mass of the original hydraulic lime binder. Commercially available graphene nanoplatelets were incorporated at the same dosage levels to provide an additional basis for comparing the effects of mineral-based and carbon-based additives. Gypsum-based plaster was also included in the initial experimental program, although its specimens were subsequently excluded from mechanical testing because of their disintegration during water curing.

All additives were incorporated as additional constituents without replacing the original hydraulic lime binder. Therefore, the hydraulic lime, water, and aggregate quantities remained constant, whereas the total powder content increased with additive dosage. Based on the mixture proportions presented in Table 1, the calculated water-to-total-powder ratios were approximately 0.350, 0.347, 0.344, and 0.340 for the reference, 1%, 2%, and 3% additive mixtures, respectively. This progressive reduction represents a mixture-proportioning effect that may influence fresh-state consistency, particle packing, and subsequent mechanical performance. Consequently, the observed strength variations cannot be attributed exclusively to the chemical or physical characteristics of the additives, since the effects of additive incorporation and changes in the water-to-total-powder ratio were not independently separated in the experimental design.

The mixture designations consisted of an abbreviation identifying the additive and a numerical value representing its percentage relative to the original hydraulic lime mass. For example, HL-1% corresponds to a mixture incorporating 1% mechanically milled hydraulic lime, whereas GN-3% denotes a mixture containing 3% graphene nanoplatelets.

The additives were introduced incrementally into the reference mixture under continuous low-speed mechanical mixing. The same general mixing procedure was adopted for both mineral additives and graphene nanoplatelets to maintain comparable preparation conditions. No ultrasonic dispersion or chemical dispersing agent was employed during mixing.

For each mixture formulation, three cubic specimens measuring 50 × 50 × 50 mm and three prismatic specimens measuring 40 × 40 × 160 mm were prepared for compressive and flexural strength testing, respectively. The freshly prepared mortar was placed into the molds in two successive layers, each compacted by 25 insertions of a steel rod to promote consistent specimen preparation.

**Figure 3:**
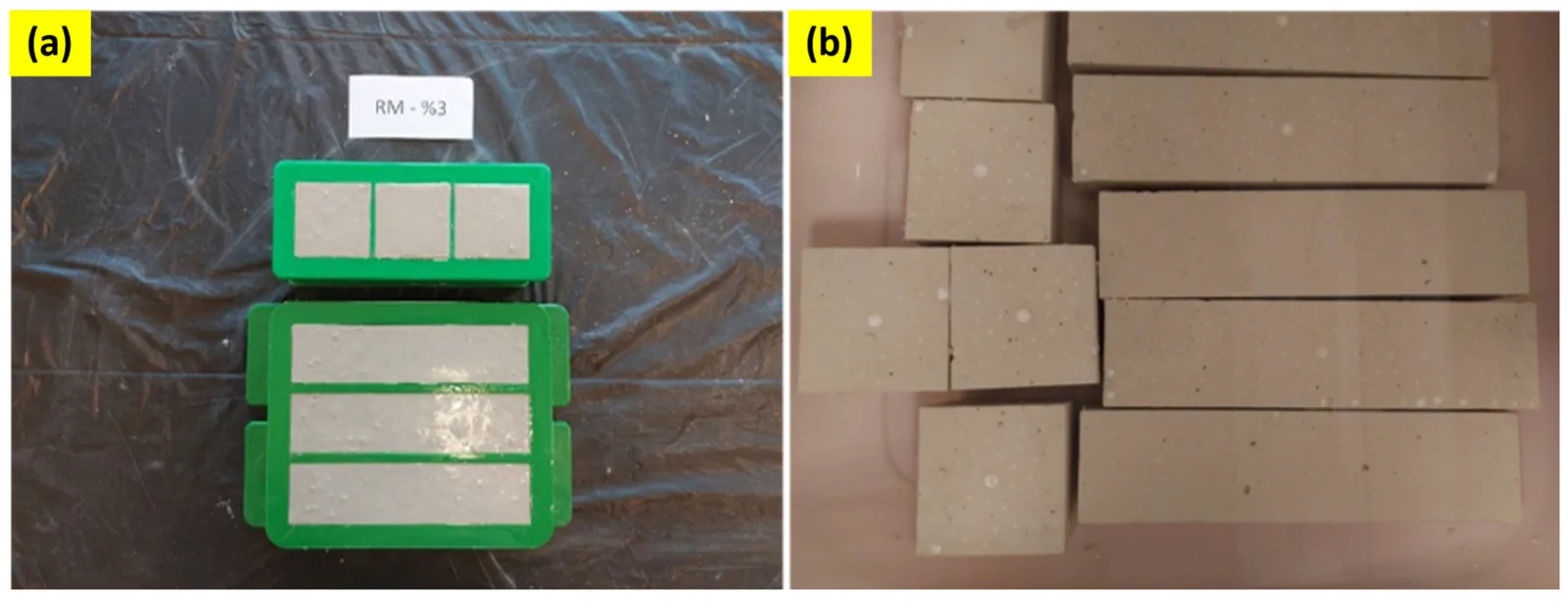
Preparation and curing of the mortar specimens: (**a**) preparation of the mortar specimens and (**b**) curing of the mortar specimens.

Following casting, the specimens were maintained in sealed molds for 48 h before demolding. Subsequently, they were immersed in water maintained at 20 ± 2 °C for 10 days. After the water-curing period, the specimens were stored under laboratory conditions at 20 ± 2 °C until mechanical testing at 28 days. The principal stages of specimen preparation and curing are illustrated in Figure 3.

### 2.3. Experimental Setups

The mechanical performance of the reference and additive-modified hydraulic lime-based mortars was evaluated through compressive and flexural strength tests conducted after 28 days of curing. The experimental program was designed to investigate the influence of additive type and incorporation level on the mechanical response of the hardened mortar specimens.

Flexural strength was determined through three-point bending tests performed on prismatic specimens measuring 40 × 40 × 160 mm. The tests followed the general principles of [25], which specify procedures for determining the flexural and compressive strengths of hardened masonry mortars. Compressive strength was evaluated using cubic specimens measuring 50 × 50 × 50 mm, with [26] adopted as the reference testing procedure.

Considering that the experimental mixtures incorporated uniformly sized porcelain spheres instead of conventional graded mortar aggregates, the mechanical testing results were interpreted within the specific material configuration employed in this study. Accordingly, the reported strengths provide a comparative assessment of the investigated mixtures rather than direct evidence of their equivalence to conventionally proportioned hydraulic lime mortars.

**Figure 4:**
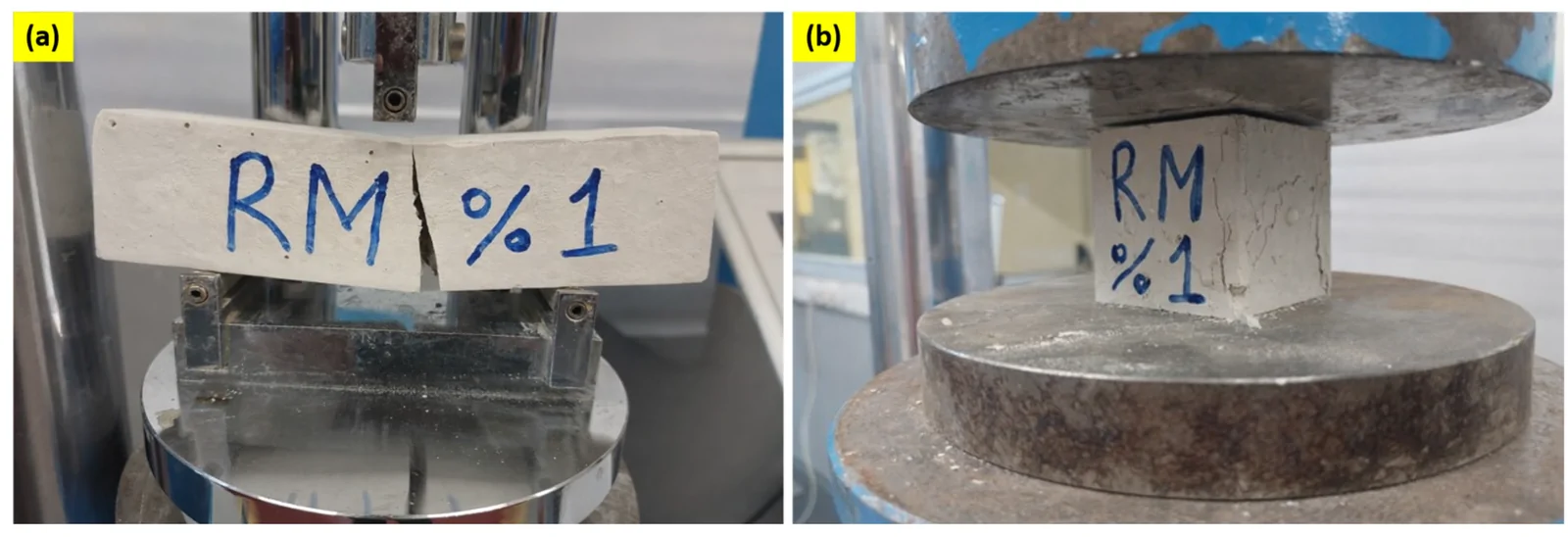
Experimental setup for the mechanical characterization of hydraulic lime-based mortar specimens: (**a**) three-point flexural strength test and (**b**) compressive strength test.

All mechanical tests were performed using a Form + Test universal testing machine with a maximum load capacity of 600 kN. The three-point flexural tests were conducted at a constant loading rate of 10 N/s, in accordance with EN 1015-11:2019 [25]. The compressive strength tests were performed at a constant loading rate of 900 N/s, following ASTM C109/C109M-24 [26]. In both tests, loading was continued until specimen failure. The experimental configurations used for flexural and compressive strength testing are presented in Figure 4.

Three specimens were tested for each mixture formulation and loading condition. The arithmetic mean of the individual measurements was adopted to represent the compressive and flexural strengths of each mixture. The resulting compressive and flexural strength values are presented in Table 2 and Table 3, respectively, and compared with those obtained from the reference mixture (PM).

**Figure 5:**
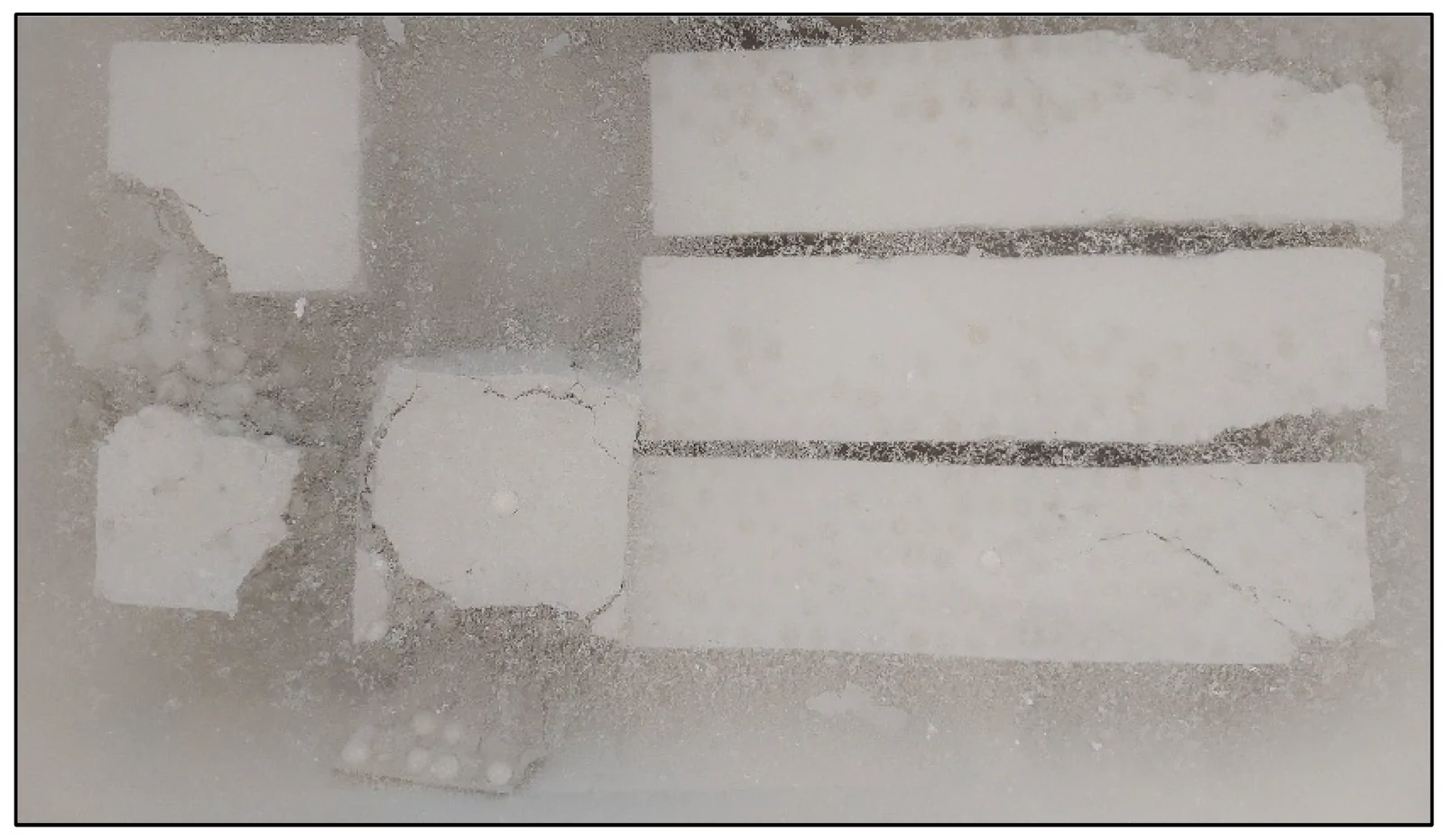
Disintegration of gypsum-containing specimens during water curing.

Gypsum-based plaster (GP) was initially included among the mineral additives subjected to mechanical milling. However, specimens incorporating GP exhibited severe deterioration and completely disintegrated during the water-curing stage following demolding, as illustrated in Figure 5. Consequently, these specimens could not be subjected to the subsequent mechanical strength tests.

The observed disintegration may be associated with changes in the hydration and phase stability of the gypsum-containing mixtures under prolonged water exposure. In particular, interactions between sulfate-bearing constituents and available calcium aluminate phases could potentially contribute to expansive reaction products, including ettringite, under suitable chemical conditions. Such reactions may result in localized expansion, microcracking, and loss of material cohesion. However, neither the formation of ettringite nor its contribution to the observed deterioration was directly verified in the present study.

Alternative mechanisms, including changes in water distribution, dissolution of susceptible constituents, and disruption of the developing binder matrix, may also have contributed to the loss of specimen integrity. Therefore, the observed behavior was considered evidence of insufficient stability of the investigated gypsum-containing formulations under the adopted curing conditions, rather than definitive confirmation of a particular chemical degradation mechanism. For this reason, GP-modified mixtures were excluded from the comparative mechanical performance analysis. The gypsum-based plaster was used as a commercially available material, and its detailed mineralogical and chemical composition was not independently characterized before incorporation into the mixtures. Consequently, the relative contributions of gypsum dissolution, sulfate-related reactions, and other moisture-induced deterioration processes could not be established. The adopted curing procedure involved immersion in water for 10 days, which may have been unfavorable for the stability of the gypsum-containing formulations. Alternative curing regimes, such as controlled-humidity curing without direct water immersion, may reduce prolonged exposure to liquid water and should be investigated in future studies. However, the effectiveness of such regimes was not evaluated in the present experimental program. Therefore, the observed disintegration should be regarded as a limitation of the investigated GP-modified mixtures under the specific curing conditions rather than evidence that gypsum-containing hydraulic lime mortars are inherently unsuitable under all curing conditions.

### 2.4. Microstructural and Compositional Characterization

Microstructural and compositional characterization was performed to investigate the morphological, elemental, mineralogical, and spectroscopic characteristics of the reference and modified hydraulic lime-based mortar mixtures. The analyses included the reference mortar (PM) and the formulations incorporating mechanically milled hydraulic lime (HL), brick powder (BP), repair mortar (RM), injection mortar (IM), slaked construction lime (SCL), and commercially available graphene nanoplatelets (GN) at addition levels of 1%, 2%, and 3%.

Three complementary analytical techniques were employed: scanning electron microscopy coupled with energy-dispersive X-ray spectroscopy (SEM-EDS), X-ray diffraction (XRD), and Fourier-transform infrared spectroscopy (FT-IR). These techniques were selected to provide information on the hardened mortar matrices and to support the interpretation of the mechanical test results.

Following flexural strength testing, representative fragments were collected from the fractured mortar specimens for microstructural and compositional examination. Before SEM analysis, the selected fragments were coated with a thin conductive gold layer to minimize charging effects and improve imaging conditions.

Scanning electron microscopy was performed using an FEI Quanta FEG 250 instrument (FEI Company, Hillsboro, OR, USA). The SEM observations were used to examine the morphology of the hardened mortar matrices, including the apparent distribution of particles, local clustering, matrix continuity, and other observable microstructural features. Particular attention was given to differences among additive types and incorporation levels that could potentially relate to variations in mechanical behavior.

Energy-dispersive X-ray spectroscopy was employed to determine the elemental composition of selected regions within the mortar matrices. The EDS results were used to identify local variations in elemental distribution associated with the different constituents. However, because EDS measurements represent localized analytical regions, the resulting elemental compositions were not considered representative of the bulk chemical composition of the entire mortar specimens.

The crystalline characteristics of the hardened mortar matrices were investigated using a Bruker D8 Advance X-ray diffractometer (Bruker AXS SE, Karlsruhe, Germany). XRD analysis was employed to identify the principal crystalline phases and compare their occurrence among the reference and modified mortar formulations. The resulting diffraction patterns provided a basis for examining similarities and differences in the mineralogical characteristics of the mixtures, particularly those associated with carbonate- and silicate-bearing constituents.

Fourier-transform infrared spectroscopy was performed using a Bruker ALPHA spectrometer (Bruker Optics GmbH & Co. KG, Ettlingen, Germany). The FT-IR analyses were conducted to identify characteristic vibrational bands associated with the constituent materials and hardened binder matrices. The resulting spectra were compared to investigate possible differences in the principal chemical bonding characteristics among the investigated formulations.

The combined SEM-EDS, XRD, and FT-IR results were evaluated alongside the measured compressive and flexural strengths to investigate potential relationships between the observed characteristics of the hardened matrices and their mechanical performance. The interpretation emphasized comparative differences among the investigated formulations rather than assigning the measured mechanical responses to a single microstructural mechanism.

It should be recognized that the analytical techniques employed in this study do not independently establish the quantity or mechanical contribution of individual hydration products, nor do they provide direct measurements of bulk porosity or pore size distribution. Furthermore, the microstructural characterization was performed on hardened mortar specimens rather than on the mineral powders before and after milling. Consequently, the analyses cannot independently verify the extent of particle refinement or structural modification resulting from the mechanical milling. These limitations were considered when interpreting the observed relationships between additive incorporation, microstructural characteristics, and mechanical performance.

## 3. Results and Discussion

### 3.1. Compressive Strength

**Figure 6:**
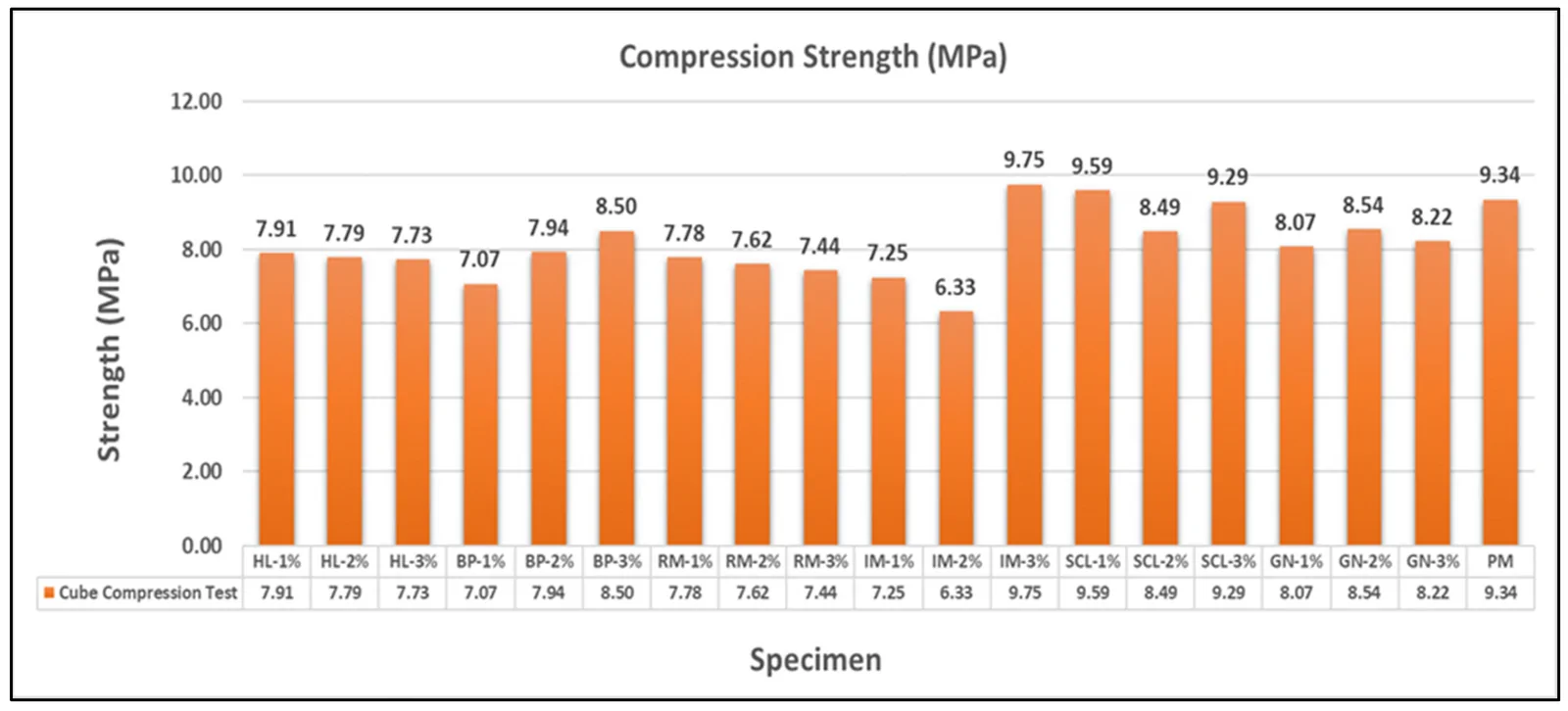
Effect of additive type and dosage on the 28-day compressive strength of hydraulic lime-based mortars.

The compressive strength results of the reference and modified hydraulic lime-based mortars after 28 days of curing are presented in Table 2 and Figure 6. The reference mortar (PM) exhibited a mean compressive strength of 9.34 MPa, which was adopted as the baseline for evaluating the effects of the different additives and incorporation levels. The results revealed considerable variations in compressive performance depending on the additive composition and dosage. Among the investigated formulations, only IM-3% and SCL-1% exceeded the reference strength, while the remaining mixtures exhibited reductions of varying magnitudes.

The incorporation of mechanically milled hydraulic lime (HL) resulted in a progressive reduction in compressive strength. The measured values were 7.91, 7.79, and 7.73 MPa for HL-1%, HL-2%, and HL-3%, respectively, corresponding to reductions of 15.3%, 16.6%, and 17.2% relative to the reference mortar. Although the differences among the three formulations were relatively limited, the results indicate that the addition of mechanically treated hydraulic lime did not improve compressive resistance under the investigated conditions. The observed response may be associated with changes in particle packing, binder distribution, or the development of local heterogeneities within the hardened matrix. However, the available experimental evidence does not permit these possible contributions to be quantified individually.

A similar reduction in compressive performance was observed for the repair mortar (RM) series. Compressive strength decreased from 7.78 MPa at 1% addition to 7.62 and 7.44 MPa at 2% and 3%, respectively. These values correspond to reductions of 16.7%, 18.4%, and 20.3% compared with PM. The consistent decline suggests that increasing the proportion of the mechanically treated repair mortar was unfavorable to compressive performance within the investigated dosage range. Although the SEM observations revealed local differences in particle arrangement and matrix continuity, they did not establish a direct relationship between the observed morphology and the progressive reduction in strength. Furthermore, the incorporation of the RM additive was not associated with clearly identifiable new crystalline phases in the XRD results.

In contrast, brick powder (BP) exhibited a progressively increasing compressive strength with increasing additive content. The mean strengths were 7.07, 7.94, and 8.50 MPa at addition levels of 1%, 2%, and 3%, respectively. Although these values remained 24.3%, 15.0%, and 9.0% below the reference strength, the reduction became progressively smaller with increasing BP content. This response suggests that the higher incorporation levels were more favorable within the BP series, although none of the investigated formulations surpassed the reference mortar. The presence of silica-bearing constituents in brick-derived materials may influence particle interactions and the development of the hardened binder matrix. However, the XRD and SEM-EDS observations did not provide direct evidence that the increasing strength resulted from enhanced pozzolanic activity. Previous investigations have similarly demonstrated that the mechanical effectiveness of mineral additions depends on their composition and interaction with the hydraulic lime binder. [9], for example, reported different strength responses for hydraulic lime-based grouts incorporating silica fume and fly ash. These findings emphasize that the incorporation of mineral materials does not necessarily result in improved mechanical performance and that additive-specific behavior must be evaluated experimentally. The mechanical response of the brick powder (BP) mixtures may involve both physical and chemical contributions, although their relative importance cannot be established from the present results. Brick powder can potentially act as a fine particulate constituent within the hydraulic lime matrix, modifying local particle arrangement and matrix continuity. Depending on its mineralogical composition and reactivity, a pozzolanic contribution is also possible. However, the XRD and FT-IR observations did not provide conclusive evidence of additional reaction products attributable specifically to BP incorporation. Moreover, no direct pozzolanic activity measurements, quantitative phase analysis, or pore-structure measurements were performed. Therefore, the observed mechanical behavior cannot be attributed definitively to either a filler effect or pozzolanic reactions. These mechanisms should be regarded as possible explanations rather than experimentally confirmed causes of the measured strength variations.

The injection mortar (IM) series exhibited a pronounced non-monotonic response in compressive strength. The mean strengths of IM-1% and IM-2% were 7.25 and 6.33 MPa, corresponding to reductions of 22.4% and 32.2%, respectively, relative to the reference mortar. Increasing the additive content to 3% resulted in a mean compressive strength of 9.75 MPa, representing a 4.4% increase over the reference and a 54.0% increase relative to IM-2%. This substantial change between the two higher dosages indicates that the mechanical response was not governed by a simple proportional relationship with additive content.

The SEM observations provide qualitative context for this behavior. IM-1% exhibited relatively large and irregular particle assemblages, while IM-2% contained more separated clusters and locally distinguishable interparticle regions. In contrast, selected regions of IM-3% displayed comparatively interconnected and compact morphological features. Such differences may be consistent with variations in local matrix continuity, particle packing, or constituent interactions. However, the available SEM images were not subjected to quantitative image analysis, and the observed surface features cannot be used to establish bulk porosity or a causal relationship with compressive strength. Additionally, the XRD and FT-IR results did not identify a distinct reaction product that could independently explain the improvement at 3% addition. The observed non-monotonic response therefore remains an experimentally established trend whose underlying mechanism requires further investigation through quantitative microstructural characterization and additional replicate testing.

The slaked construction lime (SCL) mixtures exhibited compressive strengths relatively close to that of the reference mortar. SCL-1% achieved 9.59 MPa, representing an increase of 2.7%, while SCL-2% and SCL-3% developed strengths of 8.49 and 9.29 MPa, respectively. These correspond to reductions of 9.1% and 0.5% relative to PM. The highest mean strength within the series was obtained at the lowest incorporation level, indicating that increasing the SCL dosage did not produce a progressive improvement in compressive performance.

The non-monotonic response of the SCL series may reflect differences in local particle distribution, binder interactions, or the development of the hardened matrix. Previous investigations of lime-based mortars incorporating calcium hydroxide- and silica-based nanoparticles have demonstrated that changes in additive composition and dosage can influence mechanical behavior through several interacting processes [13]. However, because the present study did not quantify particle size distributions, pore structure, or the extent of carbonation, the mechanisms responsible for the observed SCL response cannot be determined conclusively.

The incorporation of graphene nanoplatelets (GN) resulted in compressive strengths below that of the reference mortar at all investigated dosages. The recorded values were 8.07 MPa for GN-1%, 8.54 MPa for GN-2%, and 8.22 MPa for GN-3%, corresponding to reductions of 13.6%, 8.6%, and 12.0%, respectively. Although GN-2% exhibited the highest compressive strength within the graphene series, none of the formulations produced a net improvement relative to PM.

The reduction in compressive resistance may be associated with the heterogeneous distribution of graphene nanoplatelets and their interactions with the surrounding hydraulic lime matrix. In particular, the absence of ultrasonic dispersion or chemical dispersing agents during mixture preparation may have affected the uniformity of graphene distribution. However, the SEM observations did not independently establish the dimensions, concentration, or distribution of graphene agglomerates. Consequently, the observed strength reductions cannot be attributed exclusively to nanoplatelet clustering or deficient stress transfer.

A different response was reported by [23], who observed improvements in the compressive strength of hydraulic lime-based mortars containing graphene nanoplatelets at addition levels between 0.1% and 0.5%. In the present study, the graphene contents ranged from 1% to 3%, and all three mixtures exhibited lower compressive strengths than the reference mortar. The difference between these findings may be related to the higher addition levels investigated here, as well as differences in binder composition, aggregate characteristics, and graphene dispersion. However, the available experimental evidence does not allow the contribution of each factor to be isolated. The comparison therefore points to the need for further investigations covering lower graphene contents and controlled dispersion procedures.

Overall, the compressive strength results demonstrate that the mechanical response of the modified hydraulic lime-based mortars was strongly dependent on additive type and incorporation level. The HL and RM series exhibited progressive reductions with increasing dosage, whereas the BP series showed gradual improvements without exceeding the reference strength. The IM formulations exhibited the largest non-monotonic variation, with IM-3% producing the highest mean compressive strength. SCL-1% also marginally exceeded the reference strength, while graphene nanoplatelet incorporation did not improve compressive resistance at any investigated dosage.

These findings indicate that the application of mechanical milling to mineral additives does not necessarily lead to improved mechanical performance after their incorporation into hydraulic lime-based mortars. Furthermore, since untreated additive control mixtures were not investigated, the observed differences cannot be attributed exclusively to the milling procedure. Instead, they should be considered the combined response of additive composition, dosage, mixture proportions, and the resulting hardened matrix characteristics.

### 3.2. Flexural Strength

**Figure 7:**
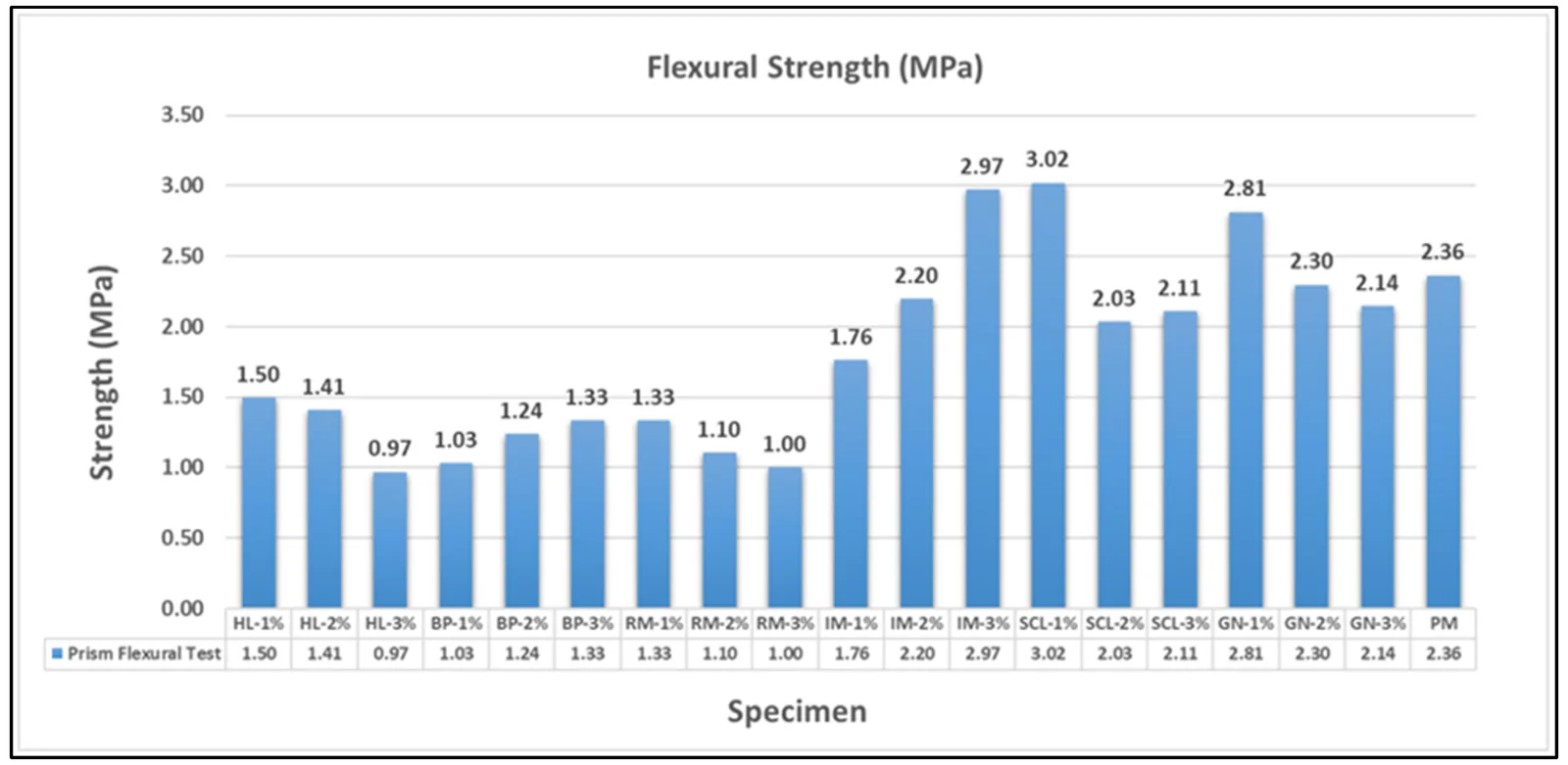
Effect of additive type and dosage on the 28-day flexural strength of hydraulic lime-based mortars.

The flexural strength results obtained from three-point bending tests after 28 days of curing are presented in Table 3 and Figure 7. The reference mortar (PM) exhibited a mean flexural strength of 2.36 MPa. Similarly to the compressive strength results, the incorporation of mineral additives and graphene nanoplatelets produced different responses depending on additive type and dosage. However, the flexural performance trends were not always consistent with those observed under compression, demonstrating that additive incorporation affected the two mechanical properties differently.

The addition of mechanically milled hydraulic lime (HL) resulted in a continuous reduction in flexural strength. The measured values were 1.50, 1.41, and 0.97 MPa for HL-1%, HL-2%, and HL-3%, respectively, corresponding to reductions of 36.4%, 40.3%, and 58.9% relative to the reference mortar. HL-3% exhibited the lowest mean flexural strength among all investigated formulations. The corresponding compressive strengths were 7.91, 7.79, and 7.73 MPa, representing reductions of 15.3%, 16.6%, and 17.2%, respectively, relative to the reference value of 9.34 MPa. Thus, although both mechanical properties deteriorated with increasing HL content, the relative reduction in flexural strength was substantially greater, particularly at 3% addition. This difference suggests that flexural resistance was more sensitive to the changes introduced by HL incorporation under the investigated conditions. Possible contributing factors include local matrix heterogeneity, changes in particle interactions, and mechanically weak regions affecting crack initiation and propagation, although these mechanisms were not directly quantified. Furthermore, because the mechanically milled hydraulic lime was added to the original binder rather than used as a replacement, increasing its dosage also altered the total powder content and water-to-total-powder ratio. Consequently, the observed deterioration cannot be attributed exclusively to the added hydraulic lime or to the mechanical milling process.

Brick powder additions exhibited a gradual increase in flexural strength with increasing dosage. The recorded values were 1.03, 1.24, and 1.33 MPa for BP-1%, BP-2%, and BP-3%, respectively. Despite this improvement within the BP series, all three formulations remained substantially below the reference mortar. Even at 3% addition, the flexural strength was approximately 43.6% lower than PM. The simultaneous increase in compressive and flexural strengths with increasing BP content suggests a favorable dosage-dependent response within the investigated series. However, the results do not establish that brick powder incorporation improved overall mechanical performance compared with the unmodified mortar.

The repair mortar (RM) series also exhibited decreasing flexural strengths with increasing additive content. The measured values were 1.33, 1.10, and 1.00 MPa at 1%, 2%, and 3% addition, respectively, corresponding to reductions of 43.6%, 53.4%, and 57.6% relative to PM. This progressive reduction was consistent with the compressive strength results, suggesting that the investigated RM additions adversely affected both mechanical properties. Although differences in particle arrangement and matrix morphology were observed, the available microstructural measurements did not establish a unique explanation for the reduction in mechanical resistance.

A substantially different response was obtained for the injection mortar (IM) series. Flexural strength increased progressively from 1.76 MPa at 1% addition to 2.20 MPa at 2% and 2.97 MPa at 3%. While IM-1% and IM-2% remained below the reference strength, IM-3% exceeded PM by approximately 25.8%, achieving the second-highest mean flexural strength among the investigated mixtures.

The favorable flexural performance of IM-3% was accompanied by the highest mean compressive strength recorded in the study. This combined response makes IM-3% one of the most promising formulations within the investigated experimental system. However, the substantial difference between IM-2% and IM-3% in compressive resistance suggests that the effects of IM incorporation are not governed by a simple linear dosage relationship. The relatively compact and interconnected features observed in selected SEM regions of IM-3% may be consistent with a more favorable local matrix organization, although quantitative measurements of porosity, particle distribution, and interfacial characteristics would be required to establish the underlying strengthening mechanism.

The slaked construction lime (SCL) series produced the highest mean flexural strength at the lowest incorporation level. SCL-1% achieved 3.02 MPa, corresponding to an improvement of 28.0% compared with PM. Increasing the dosage to 2% reduced the flexural strength to 2.03 MPa, followed by a slight increase to 2.11 MPa at 3%. Both higher-dosage mixtures remained below the reference strength, indicating that the favorable mean response was restricted to the lowest investigated SCL content.

The presence of calcium hydroxide in slaked lime may influence the local matrix structure through possible carbonation reactions and interactions with the hydraulic binder. Changes in particle arrangement may also contribute to the observed behavior. However, the available SEM-EDS, XRD, and FT-IR results do not establish a direct causal relationship between these mechanisms and the measured flexural strength, and the extent of carbonation was not quantified.

Also, the variability of the SCL-1% results requires particular consideration. As presented in Table 4, this formulation exhibited a standard deviation of 0.89 MPa and a coefficient of variation of 29.3%, indicating substantial scatter among the three tested specimens. Its mean flexural strength was also only slightly higher than that of IM-3% (2.97 MPa). The individual flexural strength values recorded for the three SCL-1% specimens were 2.11, 3.88, and 3.07 MPa. Their relatively wide spread is consistent with the coefficient of variation reported in Table 4 and highlights the uncertainty associated with the mean strength obtained from only three specimens. Consequently, SCL-1% should be regarded as the mixture with the highest observed mean flexural strength rather than a statistically established optimum, and its apparent superiority requires further experimental verification.

Graphene nanoplatelets exhibited a distinctive response under flexural loading. GN-1% achieved a mean flexural strength of 2.81 MPa, representing an improvement of 19.1% compared with the reference mortar. However, increasing the graphene content to 2% and 3% reduced flexural strength to 2.30 and 2.14 MPa, respectively. The favorable response observed at the lowest incorporation level was therefore not maintained at higher dosages.

An important observation is that GN-1% exhibited improved flexural strength despite a reduction in compressive strength from 9.34 MPa for the reference mortar to 8.07 MPa, corresponding to a decrease of approximately 13.6%. This contrasting behavior suggests that graphene nanoplatelets may influence the mechanisms governing flexural and compressive resistance differently. Under flexural loading, potential interactions between nanoplatelets and developing microcracks, including crack bridging or crack deflection, could contribute to the observed improvement. The orientation of nanoplatelets relative to the loading direction and their interaction with the surrounding binder matrix may also influence stress transfer. Conversely, nonuniform nanoplatelet dispersion, local agglomeration, or associated matrix heterogeneity could adversely affect compressive resistance. However, the present study did not directly characterize crack bridging, nanoplatelet orientation, dispersion uniformity, or interfacial stress transfer. Therefore, these mechanisms remain possible explanations rather than experimentally verified causes of the observed mechanical response.

The reduction in flexural strength at higher GN contents may also be associated with less uniform nanoplatelet distribution and localized microstructural heterogeneity, although these effects were not quantitatively established. Similar dosage-dependent behavior has been reported for other fine and nanoscale additives incorporated into lime-based systems. Reference [19] demonstrated that nano-silica and nano-alumina could produce different mechanical responses depending on the characteristics of the lime-based binder, while ref. [13] reported that nanoparticle additions could modify microstructural characteristics without necessarily increasing compressive strength. Although these studies involved different material systems, they support the broader observation that the mechanical influence of fine additives depends on matrix composition, additive characteristics, and loading conditions.

Considering all formulations, SCL-1%, IM-3%, and GN-1% exhibited the highest mean flexural strengths of 3.02, 2.97, and 2.81 MPa, respectively. In contrast, the HL, BP, and RM series remained below the reference strength at every investigated dosage. The results demonstrate that favorable flexural performance was achieved only for specific additive-dosage combinations and that increasing additive content did not necessarily produce improvements.

The experimental variability of the mechanical results was evaluated using standard deviation (SD) and coefficient of variation (CoV), as summarized in Table 4. The statistical parameters were calculated from three specimens for each mixture and loading condition. The results indicate that the degree of variability depended on both the additive type and the mechanical property being investigated. Most formulations exhibited relatively low or moderate coefficients of variation, suggesting reasonable consistency among the tested specimens under the adopted experimental procedure. However, some mixtures exhibited more pronounced variability, particularly SCL-1% in flexural strength and selected HL and BP formulations in compression. These differences highlight the importance of considering the experimental scatter when comparing relatively small changes in mean strength. For example, the increases in mean compressive strength recorded for IM-3% and SCL-1% were limited to 4.4% and 2.7%, respectively. Although these formulations exceeded the reference mean, the available statistical parameters do not independently establish that the differences are statistically significant. Furthermore, the limited number of specimens restricts the precision with which small strength differences can be evaluated.

Accordingly, the mechanical results are interpreted primarily in terms of measured mean values, observed dosage trends, and experimental variability, without assuming statistical significance where it has not been demonstrated. Additional replicate testing and inferential statistical analysis would be beneficial for confirming the reliability of the most favorable formulations.

### 3.3. Microstructural and Compositional Analysis

#### 3.3.1. SEM-EDS Analysis

**Figure 8:**
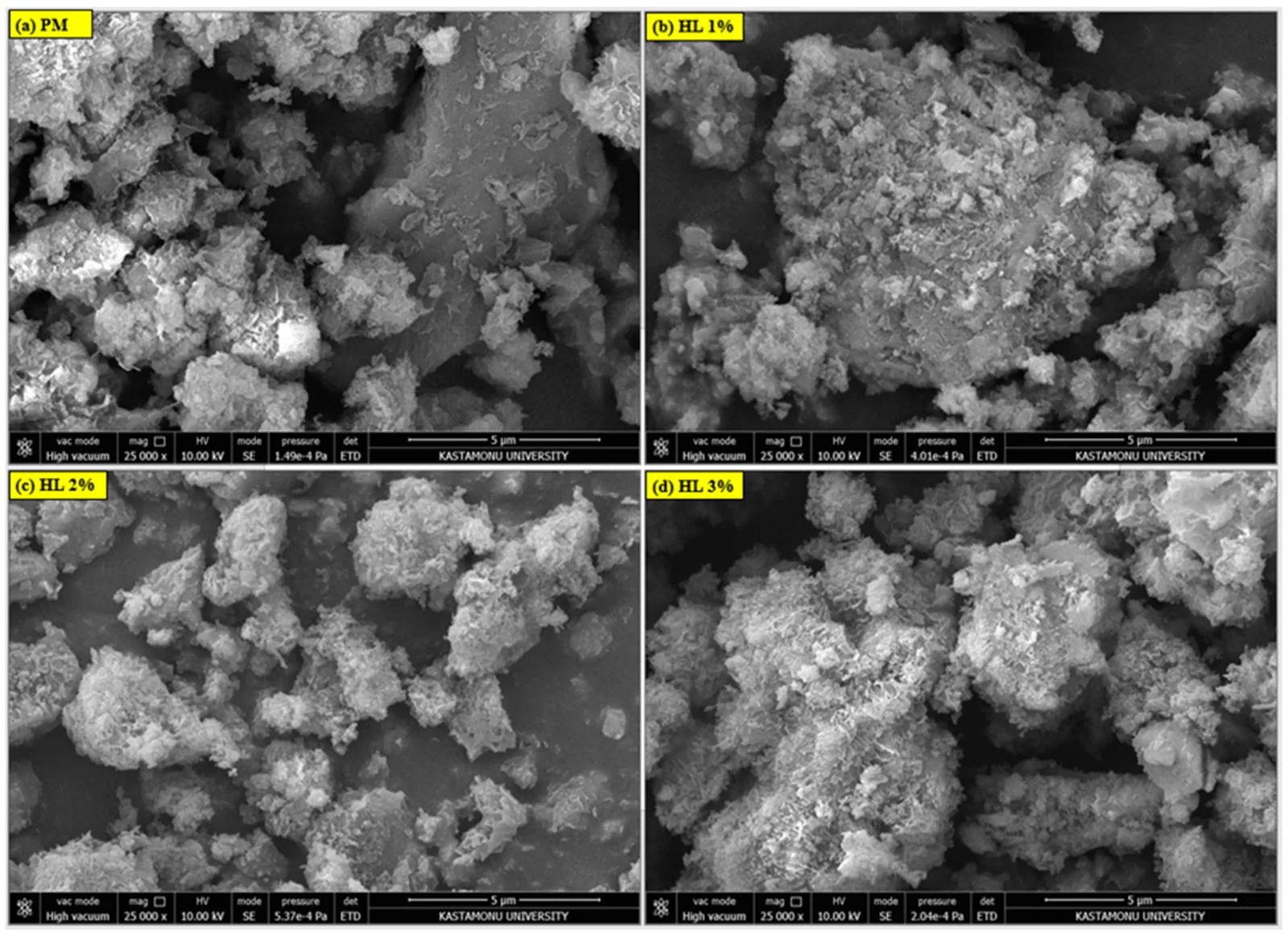
SEM micrographs of (**a**) the reference mortar (PM) and mortars incorporating mechanically milled hydraulic lime (HL) at (**b**) 1%, (**c**) 2%, and (**d**) 3%. All micrographs were acquired at a magnification of 25,000×.

**Figure 9:**
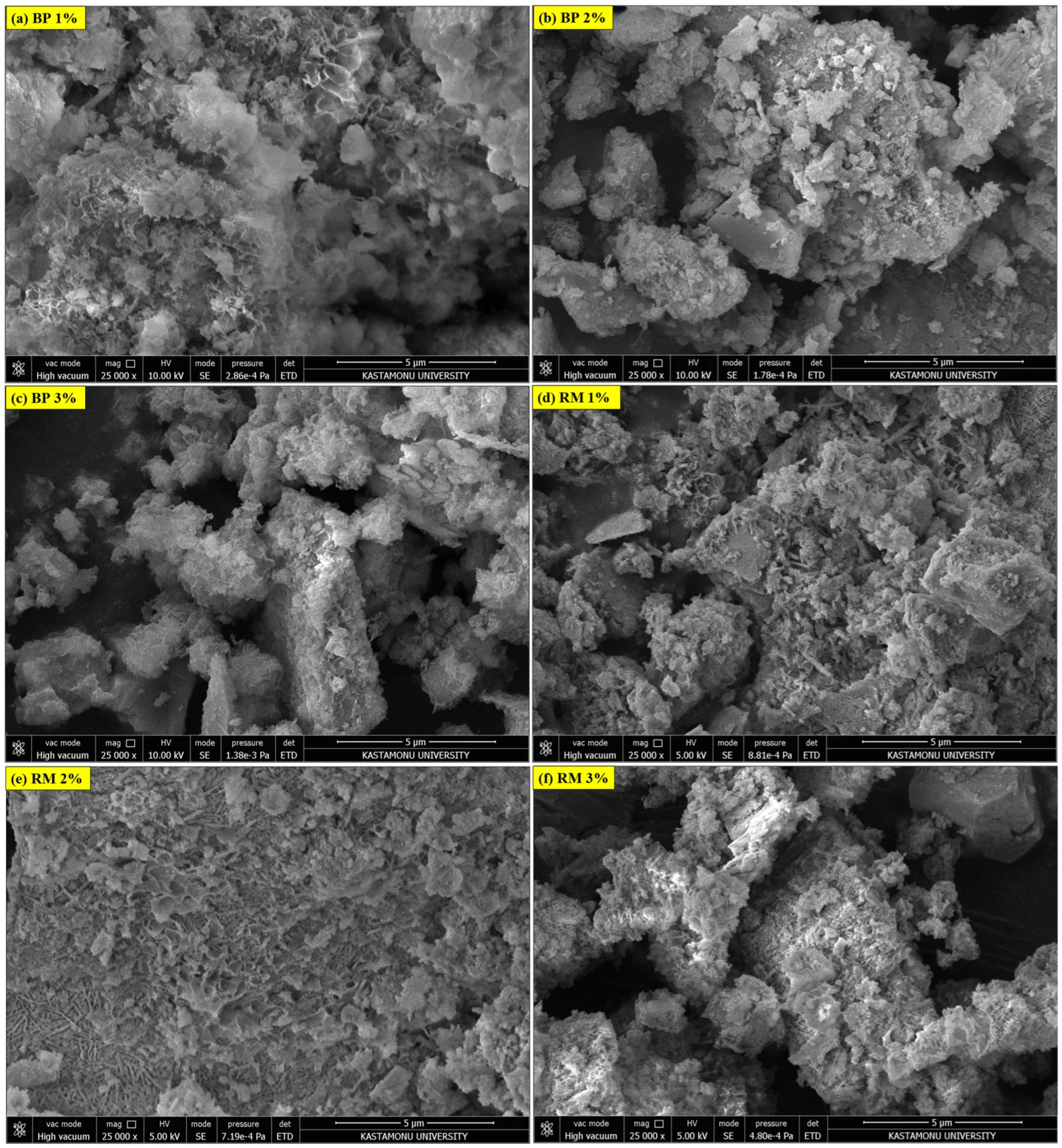
SEM micrographs of mortars incorporating mechanically milled brick powder (BP) at (**a**) 1%, (**b**) 2%, and (**c**) 3%, and repair mortar (RM) at (**d**) 1%, (**e**) 2%, and (**f**) 3%. All micrographs were acquired at a magnification of 25,000×.

**Figure 10:**
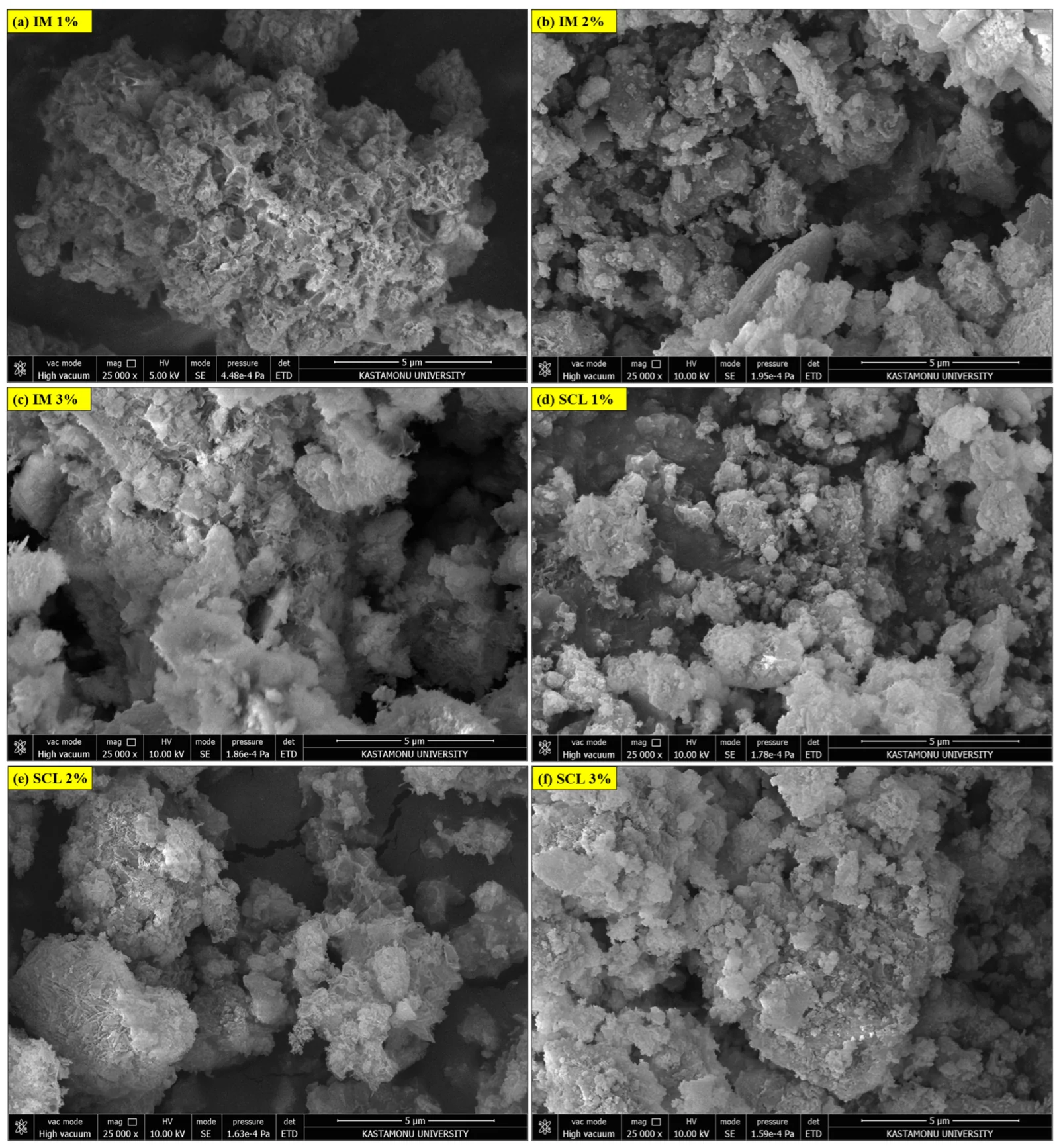
SEM micrographs of mortars incorporating mechanically milled injection mortar (IM) at (**a**) 1%, (**b**) 2%, and (**c**) 3%, and slaked construction lime (SCL) at (**d**) 1%, (**e**) 2%, and (**f**) 3%. All micrographs were acquired at a magnification of 25,000×.

**Figure 11:**
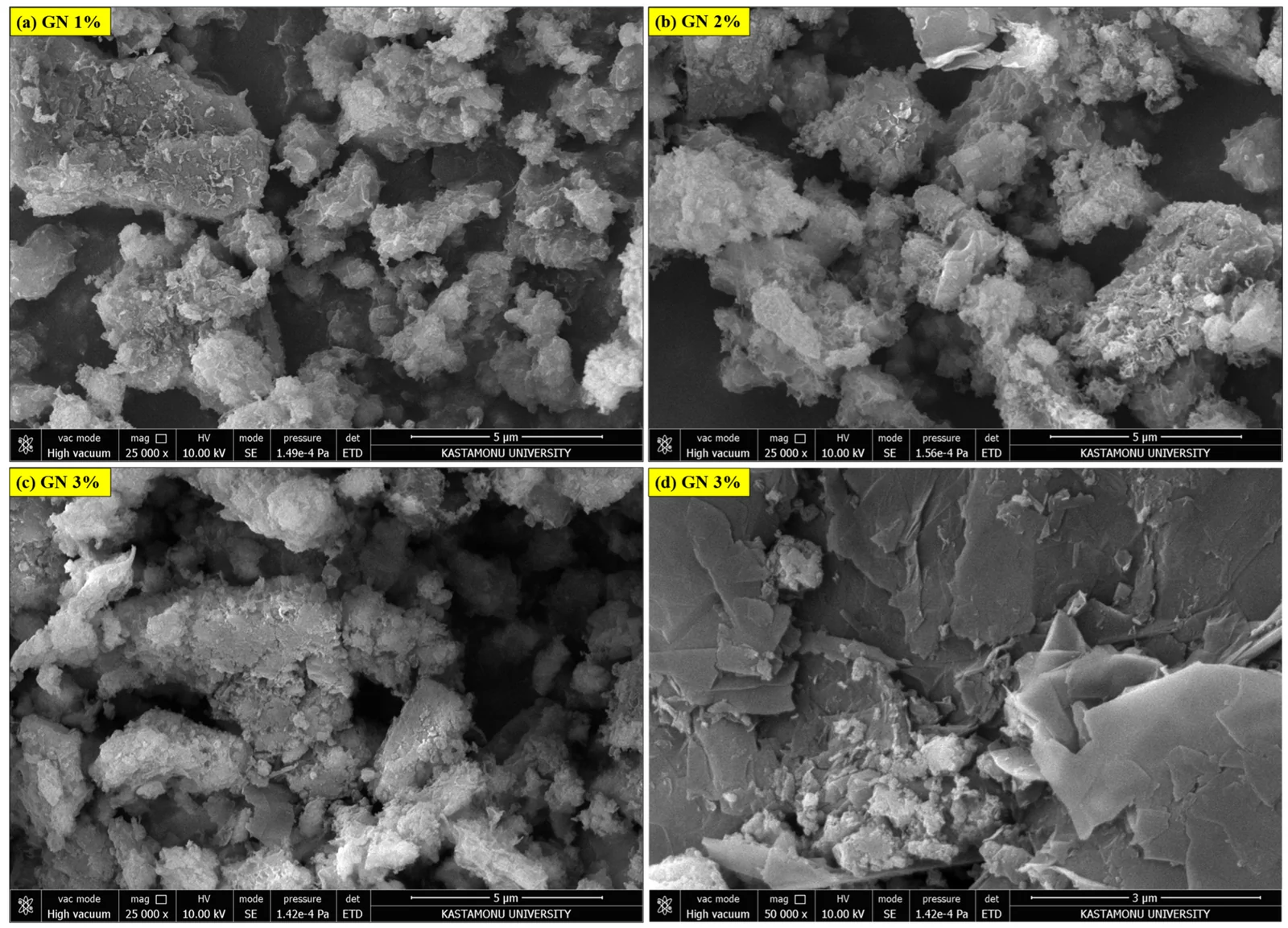
SEM micrographs of graphene nanoplatelet-modified mortars (GN) at (**a**) 1%, (**b**) 2%, and (**c**) 3%, acquired at 25,000× magnification, together with (**d**) an additional higher-magnification micrograph of GN-3% acquired at 50,000×, showing locally observed plate-like morphological features. The identity of these features as graphene nanoplatelets was not independently confirmed.

Scanning electron microscopy coupled with energy-dispersive X-ray spectroscopy (SEM-EDS) was employed to investigate the morphological characteristics and local elemental compositions of the reference and modified hydraulic lime-based mortars. The SEM micrographs presented in Figure 8, Figure 9, Figure 10 and Figure 11 reveal variations in particle arrangement, local clustering, surface morphology, and apparent matrix continuity depending on additive type and incorporation level. The reference mortar (PM) exhibited a heterogeneous matrix comprising irregular particle assemblages and relatively open interparticle regions (Figure 8). Such features provided a morphological baseline for comparing the modified formulations. The incorporation of mechanically milled mineral additives and graphene nanoplatelets resulted in changes in the visible arrangement of matrix constituents, although no consistent progression toward a more compact or homogeneous microstructure was observed with increasing additive dosage. The HL-modified mixtures exhibited differences in particle organization across the investigated dosage range (Figure 8). HL-1% contained relatively large, rough-surfaced agglomerates, whereas HL-2% showed smaller and more separated particle clusters. At 3% addition, larger irregular agglomerates became more prominent. These observations indicate that increasing the HL content did not produce a systematic improvement in apparent matrix continuity. The EDS measurements revealed a decrease in local calcium content from 62.67 wt.% for HL-1% to 36.41 wt.% for HL-3%, accompanied by an increase in silicon content from 2.14 to 6.16 wt.%. These variations reflect differences in the elemental composition of the selected analytical regions but do not independently establish the formation of additional calcium silicate hydrate phases.

The BP-modified mixtures exhibited a heterogeneous morphology characterized by irregular particulate features and locally distinguishable interparticle spaces (Figure 9). BP-1% contained fine-textured regions associated with larger particle clusters, while more pronounced particulate agglomerations were observed in BP-2%. At 3% addition, relatively coarse and angular features were evident. The EDS measurements showed local silicon contents of 4.84, 4.49, and 3.45 wt.% for BP-1%, BP-2%, and BP-3%, respectively. The absence of a progressive increase in local silicon content indicates that the improved mechanical response within the BP series cannot be explained solely by the elemental composition of the analyzed regions. Although the presence of silicon- and aluminum-bearing constituents is consistent with the mineral composition of brick-derived materials, the extent of possible pozzolanic reactions cannot be established from these observations.

The RM series exhibited noticeable differences in local morphological characteristics (Figure 9). RM-1% was characterized by coarse and irregular particle assemblages, while RM-2% displayed comparatively more continuous and interconnected surface features. At 3% addition, larger clusters and more distinguishable open regions were observed. Despite the locally more continuous appearance of RM-2%, its mechanical strengths remained below those of RM-1%. This observation demonstrates that localized morphological continuity does not necessarily correspond to improved bulk mechanical performance. The EDS results were predominantly characterized by calcium and oxygen, with smaller contributions from silicon and aluminum.

The IM-modified mixtures showed particularly distinct dosage-dependent morphological characteristics (Figure 10). IM-1% exhibited relatively large and rough-surfaced particle assemblages, whereas IM-2% contained smaller and more separated clusters. In contrast, IM-3% displayed larger interconnected regions with locally more compact features. These observations are relevant because IM-3% also exhibited the highest compressive strength and a substantial improvement in flexural strength. The EDS results further revealed differences in the elemental composition of the selected regions. IM-1% exhibited 58.58 wt.% calcium and 0.93 wt.% silicon, while IM-2% presented the highest local silicon content within the series at 6.26 wt.%. For IM-3%, the corresponding calcium and silicon contents were 42.80 and 4.33 wt.%, respectively. Interestingly, the highest local silicon concentration occurred in IM-2%, which exhibited the lowest compressive strength within the IM series. This result indicates that local elemental composition alone cannot explain the observed mechanical response. The favorable performance of IM-3% may be associated with differences in matrix organization and constituent interactions, although this interpretation requires quantitative microstructural verification.

The SCL mixtures exhibited non-monotonic changes in their morphological characteristics (Figure 10). SCL-1% contained irregular fine particle assemblages distributed around larger fragments, whereas SCL-2% exhibited comparatively coarser and smoother particle features. More pronounced clustered regions were observed in SCL-3%. The local calcium content decreased from 54.18 wt.% for SCL-1% to 43.76 wt.% for SCL-3%, while oxygen increased from 40.46 to 51.83 wt.%. Silicon remained within a relatively narrow range of 3.89–4.44 wt.%. These results indicate that the favorable mechanical response of SCL-1%, particularly under flexural loading, cannot be attributed to a simple increase in silicon-bearing constituents or to a progressive change in elemental composition with dosage.

Graphene nanoplatelet-modified mortars exhibited heterogeneous particle arrangements throughout the investigated dosage range (Figure 11). GN-1% contained fine irregular features distributed among larger matrix fragments, while more pronounced clustered regions were observed in GN-2% and GN-3%. The EDS results showed local silicon contents of 4.19, 4.72, and 2.13 wt.% for GN-1%, GN-2%, and GN-3%, respectively, while calcium increased from 33.67 to 40.37 wt.% across the same dosage range. An additional SEM micrograph of GN-3% acquired at 50,000× magnification is presented in Figure 11d to provide a closer view of locally observed plate-like morphological features. However, their identification as graphene nanoplatelets remains unconfirmed.

Although the increasingly clustered morphology observed at higher GN contents may be compatible with less uniform nanoplatelet distribution, the available SEM-EDS observations do not directly identify individual graphene nanoplatelets or quantify their dispersion, preferred orientation, or agglomeration within the mortar matrix. Also, potential crack-bridging and crack-deflection mechanisms could not be directly verified from the available micrographs. Consequently, neither the reduction in flexural strength beyond 1% GN addition nor the contrasting compressive and flexural responses of GN-1% can be attributed conclusively to these mechanisms. However, the observed local microstructural heterogeneity provides a possible qualitative context for interpreting the dosage-dependent mechanical behavior.

Overall, the SEM-EDS results demonstrate that additive incorporation influenced the local morphology and elemental characteristics of the hardened hydraulic lime-based matrices. However, increasing additive content did not lead to a uniform improvement in apparent matrix compactness or continuity. Instead, each additive exhibited a distinct morphological response, frequently characterized by non-monotonic changes with dosage.

The correspondence between selected morphological features and mechanical behavior suggests that matrix organization may contribute to the measured strength variations. However, SEM observations and localized EDS measurements cannot independently quantify bulk porosity, interfacial bonding, or the mechanical contribution of individual constituents. These limitations must be considered when relating the observed microstructures to the macroscopic mechanical properties.

#### 3.3.2. XRD Analysis

X-ray diffraction analysis was performed to investigate the crystalline characteristics of the reference and modified hydraulic lime-based mortars and to identify possible changes associated with additive incorporation.

**Figure 12:**
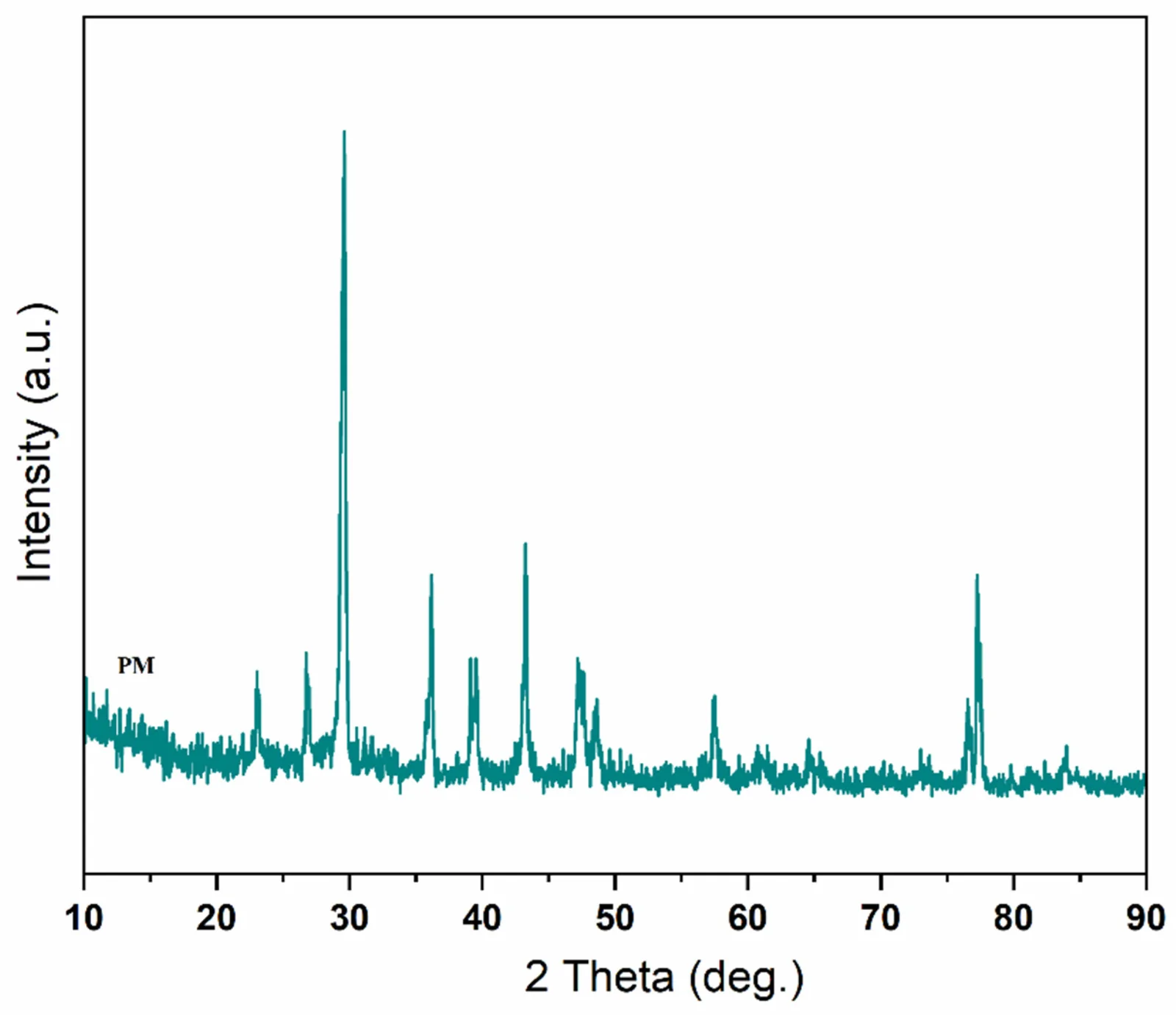
XRD pattern of the reference hydraulic lime-based mortar (PM).

The XRD pattern of the reference mortar (PM), presented in Figure 12, exhibited a dominant diffraction peak at approximately 29.4° 2θ, corresponding to an interplanar spacing of approximately 3.04 Å. Additional reflections were observed near 23°, 36°, 39°, 43°, 47–49°, and 57–61° 2θ. These reflections are consistent with a calcite-rich crystalline assemblage, indicating that carbonate-bearing phases constituted a major crystalline component of the hardened matrix. A reflection near 26–27° 2θ was also observed and is consistent with quartz or other silica-bearing crystalline constituents.

**Figure 13:**
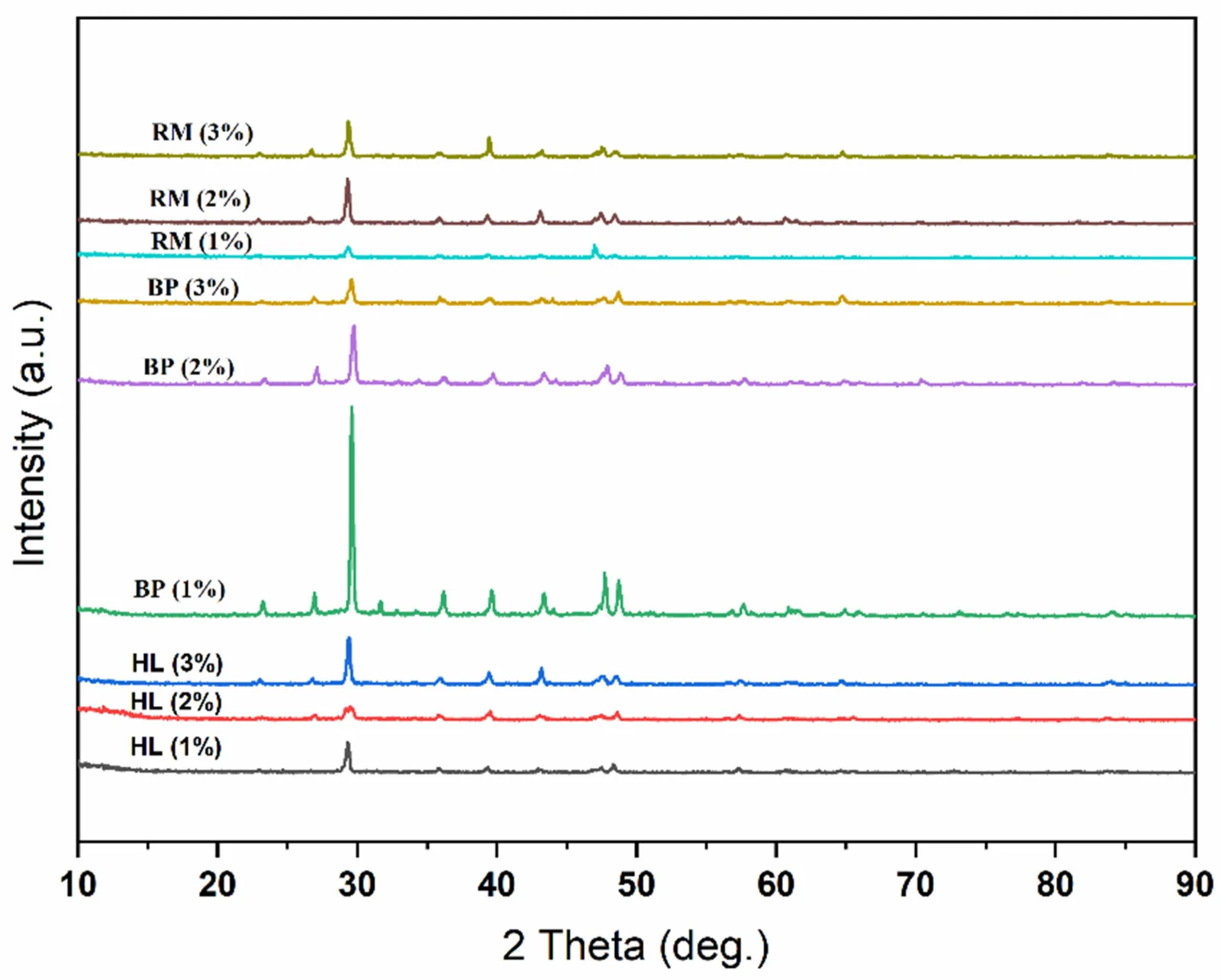
XRD patterns of HL-, BP-, and RM-modified mortars at 1%, 2%, and 3% addition levels.

The diffraction patterns of the HL-, BP-, and RM-modified mixtures are presented in Figure 13. Across these series, the dominant reflection remained within approximately 29.3–29.7° 2θ, accompanied by secondary peaks associated with carbonate- and silica-bearing crystalline constituents. The preservation of the principal peak positions indicates that the investigated additives did not produce a major change in the identifiable crystalline assemblage of the hardened hydraulic lime-based matrix.

Within the HL series, the principal reflections occurred at 29.318°, 29.478°, and 29.418° 2θ for HL-1%, HL-2%, and HL-3%, respectively. Secondary reflections near 35–36°, 39°, 43°, and 47–48° were also retained. HL-3% exhibited additional relatively weak reflections near 17.9° and 34.2° 2θ, potentially associated with residual calcium hydroxide-bearing crystalline constituents. However, these observations do not provide a quantitative measure of hydration or carbonation processes.

The BP series exhibited a comparatively pronounced reflection near 26–27° 2θ, consistent with the presence of silica-bearing crystalline constituents associated with the brick-derived additive. However, the dominant carbonate-related reflection remained near 29.4–29.7° 2θ at all investigated dosages. No systematic displacement of the principal diffraction peaks was observed with increasing BP content. Consequently, the progressive increase in mechanical strength within the BP series cannot be attributed directly to the formation of a new highly crystalline phase detectable through the available XRD measurements. Although brick powder may exhibit pozzolanic activity under suitable conditions, the present XRD patterns do not independently demonstrate the formation of additional hydration products resulting from BP incorporation. In particular, the absence of clearly identifiable new crystalline phases should not be interpreted as proof that no reaction occurred, since poorly crystalline or amorphous reaction products may not be readily resolved by conventional XRD. Accordingly, the contribution of pozzolanic reactions remains uncertain and cannot be separated from possible physical effects using the available characterization results.

The RM-modified mixtures similarly retained the principal carbonate-related reflection near 29.3–29.6° 2θ, together with secondary features around 39°, 43°, and 47–49°. Although variations in relative peak intensities were observed, the principal crystalline characteristics remained broadly comparable among the three dosages. Accordingly, the decreasing mechanical strengths of the RM series were not accompanied by the emergence of a distinct new crystalline phase.

**Figure 14:**
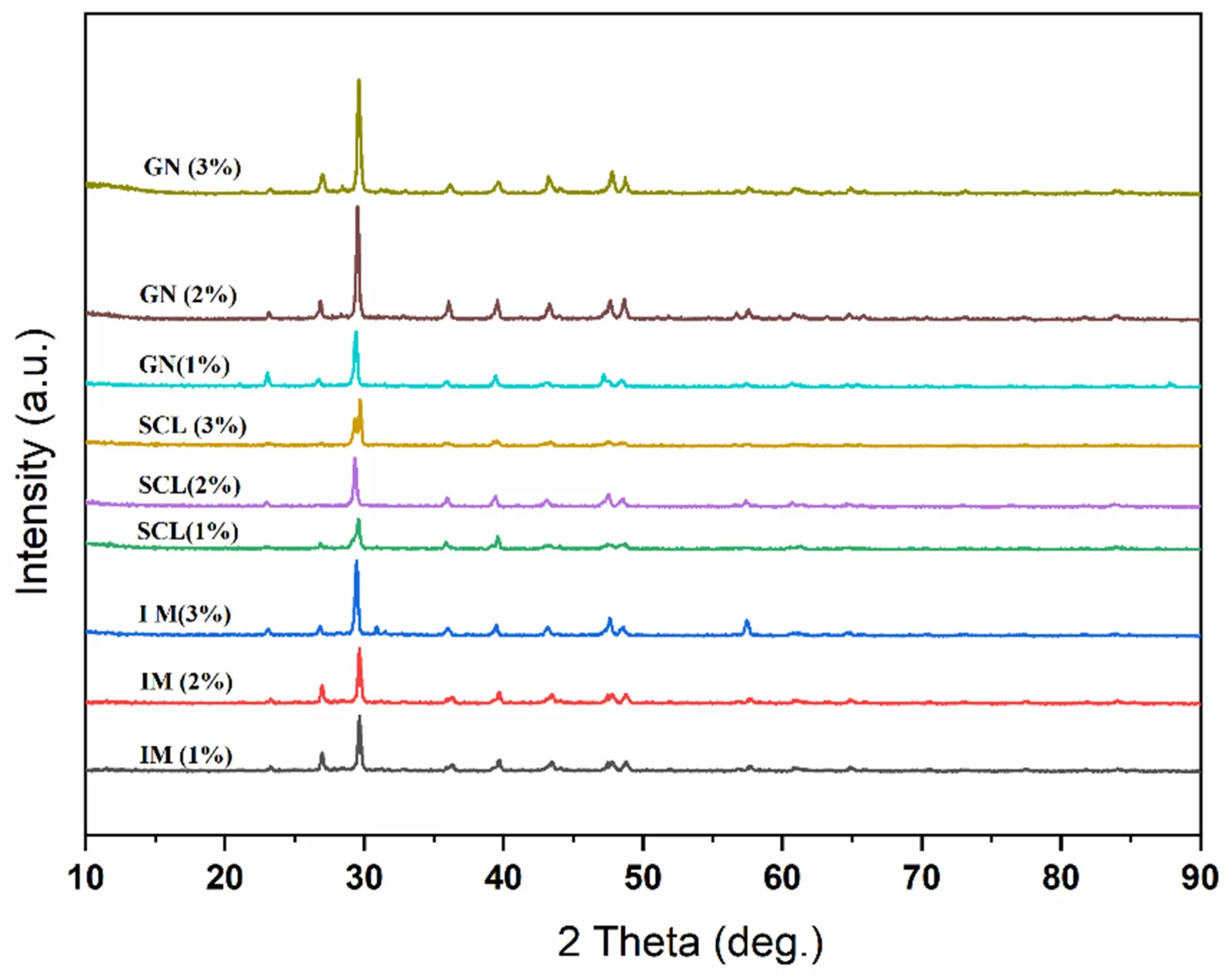
XRD patterns of IM-, SCL-, and GN-modified mortars at 1%, 2%, and 3% addition levels.

The XRD patterns of the IM-, SCL-, and GN-modified mortars are presented in Figure 14. Similarly to the other mixtures, the dominant reflections remained concentrated around 29–30° 2θ, indicating the persistence of the carbonate-rich crystalline characteristics of the reference matrix.

The IM series exhibited principal reflections near 29.4–29.6° 2θ at all incorporation levels. IM-3% also retained secondary features near 36°, 39.5°, 43.3°, and 47–49° 2θ. Importantly, the superior mechanical performance of IM-3% was not accompanied by a unique intense diffraction peak absent from the lower-dosage mixtures. This observation indicates that its higher measured strength cannot be explained solely by the appearance of a newly identifiable crystalline compound.

The SCL series exhibited similar characteristics, with the dominant reflection remaining near 29–30° 2θ and variations occurring primarily in relative peak intensities. Despite achieving the highest mean flexural strength, SCL-1% did not exhibit a unique dominant crystalline phase that could independently explain its mechanical response.

The GN-modified mixtures also retained the principal crystalline features of the hydraulic lime-based matrix. Carbonate-related reflections remained dominant, while a silica-associated feature was observed near 26–27° 2θ. No distinct new crystalline reflection attributable exclusively to graphene nanoplatelet incorporation was identified. However, the absence of such a reflection does not establish that graphene-related interactions or poorly crystalline reaction products were absent, since their identification may be limited by concentration, structural characteristics, and the sensitivity of the measurement technique.

Collectively, the XRD results indicate that the modified hydraulic lime-based mortars largely retained a calcite-rich crystalline assemblage, accompanied by silica-bearing crystalline constituents whose relative contributions varied among formulations. The principal differences were associated with changes in diffraction intensity rather than the appearance of clearly distinguishable new crystalline phases.

These findings suggest that the observed mechanical differences cannot be explained exclusively through the qualitative identification of crystalline constituents. However, the available XRD data do not quantify amorphous phases, hydration-product contents, or changes in reaction extent. Therefore, possible relationships between mineralogical characteristics and mechanical performance require complementary interpretation alongside the SEM-EDS and FT-IR observations.

#### 3.3.3. FT-IR Analysis

**Figure 15:**
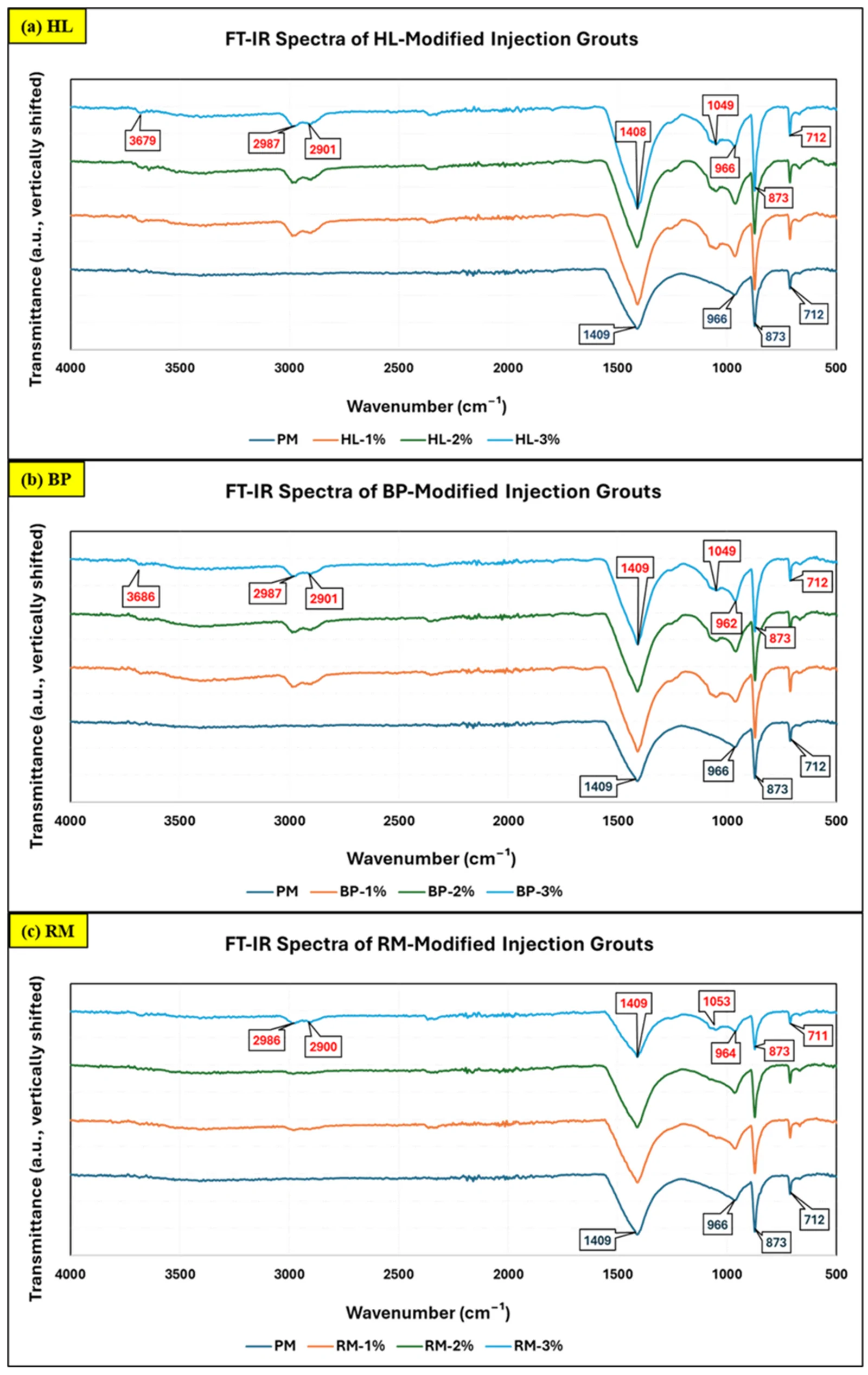
FT-IR spectra of HL-, BP-, and RM-modified hydraulic lime-based mortars at different additive contents.

**Figure 16:**
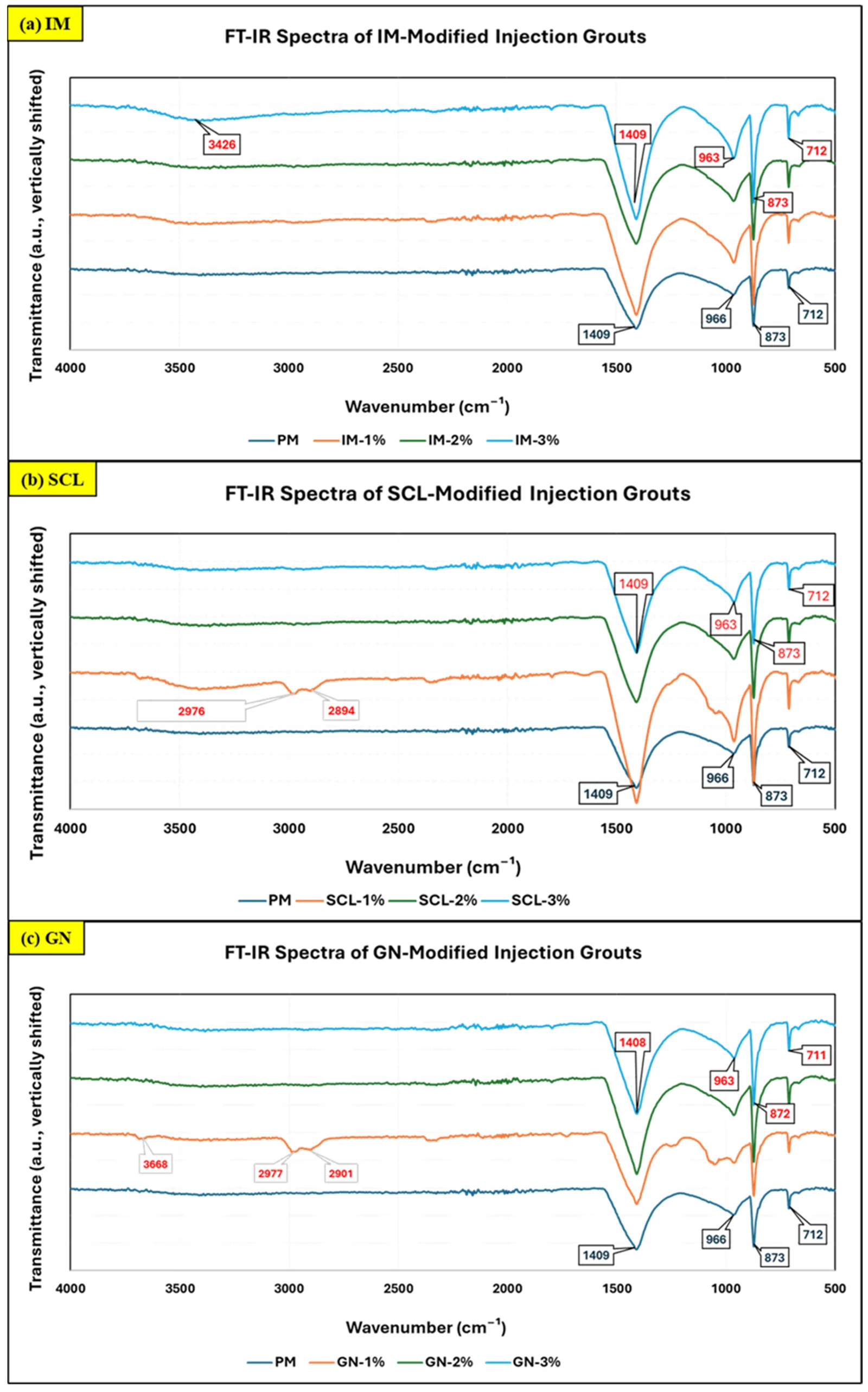
FT-IR spectra of IM-, SCL-, and GN-modified hydraulic lime-based mortars at different additive contents.

Fourier-transform infrared spectroscopy was employed to examine the characteristic vibrational bands of the reference and additive-modified hydraulic lime-based mortars. The corresponding spectra are presented in Figure 15 and Figure 16. Across the investigated formulations, prominent absorption bands were detected near 1408–1409, 872–873, and 711–712 cm^−1^. These bands are associated with characteristic carbonate-group vibrations and are consistent with the presence of calcite identified through XRD analysis. Their persistence across the different additive groups indicates that the principal carbonate-related chemical signatures of the hardened matrix remained detectable after additive incorporation. Additional absorption features were observed in the region of approximately 962–966 cm^−1^, which can be associated with Si-O vibrations in silicate- and aluminosilicate-containing environments. In hydraulic lime systems, this spectral region may also include contributions from poorly crystalline calcium silicate hydrate products. However, overlapping vibrational bands prevent the unambiguous identification or quantification of individual hydration products through FT-IR alone.

The HL-modified mixtures exhibited a band near 3679 cm^−1^, consistent with hydroxyl vibrations associated with calcium hydroxide-bearing constituents. Additional weak features were reported near 2987 and 2901 cm^−1^, while carbonate-related bands remained prominent throughout the dosage range. The absorption near 966 cm^−1^ indicates the persistence of silicate-associated bonding environments. Although differences in spectral characteristics were observed among the HL formulations, they do not independently demonstrate a progressive increase in hydration-product formation.

The BP mixtures retained the characteristic carbonate bands together with silicate-related features in the 962–966 cm^−1^ region. A hydroxyl-associated band was also reported near 3686 cm^−1^. The coexistence of carbonate- and silicate-related absorption is consistent with the incorporation of brick-derived mineral constituents into the hydraulic lime matrix. However, the spectra do not establish the extent of pozzolanic reactions or explain the progressive mechanical improvement observed within the BP series.

The RM-modified mixtures exhibited a similar carbonate-dominated spectral profile, with prominent absorption bands near 1409, 873, and 711–712 cm^−1^. Additional features around 964–966 and 1053 cm^−1^ indicate contributions from silicate- or aluminosilicate-bearing environments. Weak bands near 2986 and 2900 cm^−1^ may be associated with minor carbon-hydrogen-containing species. However, no distinct spectral characteristic was identified that could independently explain the decreasing mechanical strength with increasing RM content.

The IM-modified mixtures exhibited carbonate-related absorption bands near 1409, 873, and 712 cm^−1^, together with a silicate-associated feature at approximately 963–966 cm^−1^. IM-3% additionally exhibited a more noticeable absorption feature near 3426 cm^−1^, potentially associated with hydroxyl groups or adsorbed and chemically bound water. Although this feature may reflect differences in the moisture- or hydroxyl-related characteristics of the hardened matrix, it does not provide a quantitative measure of hydration or independently account for the higher mechanical strength of IM-3%.

The SCL-modified mixtures retained the characteristic carbonate-related bands near 1409, 873, and 712 cm^−1^. A comparatively limited silicate-associated contribution was observed around 963–966 cm^−1^. SCL-1% also exhibited weak features near 2976 and 2894 cm^−1^, potentially associated with carbon-hydrogen stretching vibrations of minor constituents. However, the spectra did not reveal a unique dominant bonding environment that could directly explain the higher flexural strength of SCL-1%.

The GN-modified mixtures similarly exhibited carbonate-related absorption bands near 1408–1409, 872–873, and 711–712 cm^−1^, together with silicate-associated features near 963–966 cm^−1^. Additional weak absorptions were reported near 3668, 2977, and 2901 cm^−1^ for GN-1%. These may reflect hydroxyl- and carbon-hydrogen-related contributions, although their assignment remains limited by the heterogeneous composition of the mortar matrix.

No clearly distinguishable new absorption bands could be assigned conclusively to newly formed reaction products resulting from graphene nanoplatelet incorporation. This observation is consistent with the qualitative XRD results, which indicated that the principal crystalline characteristics of the hydraulic lime-based matrix were retained.

Overall, the FT-IR spectra indicate that the modified mortars largely preserved the principal carbonate- and silicate-related bonding characteristics of the reference hydraulic lime-based matrix. Variations among additive types and dosages were mainly reflected in secondary absorption features rather than in the appearance of entirely new dominant spectral characteristics.

The agreement between the XRD and FT-IR results suggests that the incorporation of the investigated additives did not produce readily detectable major changes in the principal crystalline and chemical signatures of the hardened mortars. However, neither technique independently quantifies hydration products, reaction progress, or the spatial distribution of the additives. Therefore, the observed mechanical differences should be interpreted through the combined consideration of the mechanical, morphological, mineralogical, and spectroscopic results.

#### 3.3.4. Relationship Between Microstructure and Mechanical Performance

The combined mechanical and microstructural results indicate that the response of hydraulic lime-based mortars incorporating mechanically milled mineral additives and graphene nanoplatelets depended strongly on both additive composition and incorporation level. Although the modified mixtures generally retained comparable carbonate- and silicate-related crystalline and spectroscopic characteristics, their mechanical strengths varied considerably. This contrast indicates that the measured differences cannot be attributed solely to the formation of distinct new crystalline phases.

The progressive reductions observed in the HL and RM series coincided with heterogeneous particle distributions and clustered regions identified in the SEM micrographs. These morphological characteristics may have contributed to the mechanical response through changes in local matrix continuity or constituent interactions. However, the SEM observations do not provide quantitative evidence that these features were responsible for the strength reductions.

In contrast, the progressive improvement observed within the BP series may reflect more favorable interactions between the brick-derived constituents and the surrounding hydraulic lime matrix at higher incorporation levels. However, neither the XRD nor the FT-IR results confirmed the formation of additional reaction products that could directly explain this trend.

The IM-3% mixture exhibited the most favorable overall combination of compressive and flexural strength. Its mechanical response coincided with locally interconnected and comparatively compact morphological features observed by SEM. Although these observations suggest a possible relationship between matrix organization and mechanical performance, the available evidence does not establish whether differences in particle packing, pore structure, or hydration-product development were primarily responsible for the improvement.

Similarly, SCL-1% exhibited improved mean mechanical strengths, particularly in flexure, without a corresponding unique crystalline or spectroscopic characteristic. The GN-1% mixture showed an increase in flexural strength despite a reduction in compressive resistance. This response may be associated with differences in crack development and local stress transfer, although direct evidence of graphene-related crack bridging or interfacial reinforcement was not obtained.

Taken together, these findings highlight the importance of considering microstructural organization and additive-specific interactions when interpreting mechanical behavior. However, the results do not establish a universal strengthening mechanism applicable to all investigated additives. Additional characterization of particle size distributions, pore structure, additive dispersion, and hydration products would be required to distinguish the individual contributions of these factors.

### 3.4. Synthesis and Mechanistic Discussion

The experimental results demonstrate that the incorporation of mechanically milled mineral additives and graphene nanoplatelets into hydraulic lime-based mortars produced strongly composition- and dosage-dependent mechanical responses. Although selected formulations exhibited improvements relative to the reference mortar, most modified mixtures developed lower compressive and flexural strengths. Consequently, the effectiveness of additive incorporation cannot be generalized across the investigated materials.

Among the mineral additives, IM-3% exhibited the most favorable combined mechanical performance, achieving a compressive strength of 9.75 MPa and a flexural strength of 2.97 MPa. These values represent improvements of 4.4% and 25.8%, respectively, compared with the reference mortar. SCL-1% also exceeded the reference in both mechanical properties, reaching 9.59 MPa in compression and 3.02 MPa in flexure. However, the comparatively high variability of the SCL-1% flexural results limits the confidence with which its apparent superiority can be interpreted.

The GN-1% mixture exhibited a different mechanical response, with a 19.1% improvement in flexural strength accompanied by a 13.6% reduction in compressive strength. This contrast demonstrates that enhancement of one mechanical property does not necessarily imply an improvement in the overall mechanical performance of the material. The observed behavior may reflect differences in the mechanisms governing tensile stress transfer, crack initiation, and compressive failure. However, direct experimental evidence of graphene-mediated crack bridging or improved fracture resistance was not obtained.

**Figure 17:**
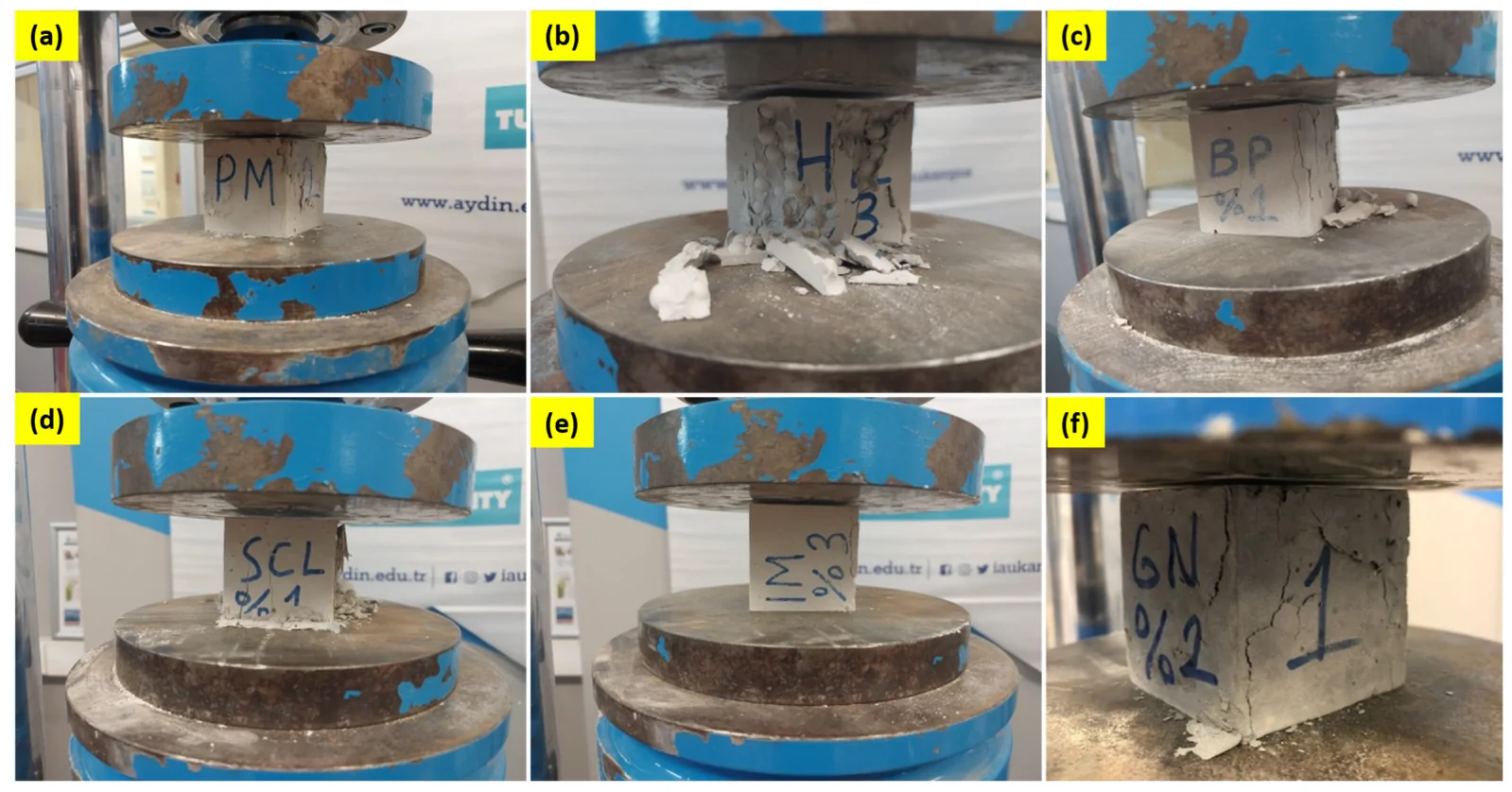
Post-failure appearance of representative cube specimens following compressive strength testing at 28 days: (**a**) PM reference mortar; (**b**) HL-3%; (**c**) BP-1%; (**d**) SCL-1%; (**e**) IM-3%; and (**f**) GN-2%.

The post-failure appearances of representative specimens, presented in Figure 17, provide additional qualitative information concerning the failure characteristics of the investigated mixtures. The reference mortar and several mineral-additive formulations exhibited relatively extensive fragmentation following compressive failure, whereas the representative GN-2% specimen retained a greater portion of its original geometry and displayed several visible cracks. Such differences suggest that the additives may influence the development and localization of cracking during failure. However, post-failure photographs alone are insufficient to establish improvements in ductility, toughness, or energy dissipation. These properties would require direct evaluation through deformation measurements and post-peak mechanical response.

The experimental findings are consistent with previous investigations demonstrating that the performance of mineral additions depends strongly on binder composition and material characteristics. Reference [9] reported different mechanical responses for hydraulic lime-based grouts incorporating silica fume and fly ash. Similarly, ref. [13] demonstrated that nanoparticle additions could modify the microstructural and deformation characteristics of lime-based mortars without necessarily improving compressive strength. Reference [19] also reported different mechanical responses to nano-silica and nano-alumina depending on the composition of the lime-based binder. These studies collectively reinforce the importance of evaluating each additive within its specific material system.

An important consideration is that the mineral additives were incorporated after planetary ball milling, but their particle size distributions and structural characteristics before and after treatment were not directly compared. Furthermore, equivalent mixtures containing untreated additives were not included in the experimental program. Consequently, the observed mechanical responses cannot be attributed specifically to the milling process. They should instead be interpreted as the combined effects of the additive materials, their applied processing conditions, their incorporation levels, and their interactions with the reference mortar formulation.

A further consideration concerns the relationship between mechanical performance and suitability for historic masonry conservation. Although increased mechanical strength may be advantageous in selected applications, compatibility with historic masonry materials requires additional consideration of elastic stiffness, deformation characteristics, moisture transport, dimensional stability, and long-term durability. In particular, the behavior of the investigated laboratory mixtures cannot be assumed to represent that of conventional lime-based injection grouts without direct assessment of fresh-state rheology, penetrability, and application-related properties.

The findings therefore identify specific additive-dosage combinations that warrant further investigation rather than establishing formulations ready for practical conservation applications. IM-3% represents the most favorable combination of measured compressive and flexural performance, while SCL-1% and GN-1% demonstrate potentially beneficial but more property-specific responses. Further research should focus on verifying the mechanical results, examining lower additive dosages, characterizing the processed powders, and evaluating the long-term physical and mechanical compatibility of the resulting mortars.

## 4. Conclusions

This study investigated the mechanical and microstructural performance of hydraulic lime-based mortars incorporating mechanically milled mineral additives and commercially available graphene nanoplatelets. Hydraulic lime, brick powder, repair mortar, injection mortar, slaked construction lime, and gypsum-based plaster were subjected to planetary ball milling, while graphene nanoplatelets were incorporated without additional mechanical treatment. The additives were introduced at 1%, 2%, and 3% by mass of the original hydraulic lime binder. The resulting mortars were evaluated through compressive and flexural strength tests at 28 days, supported by SEM-EDS, XRD, and FT-IR analyses. Based on the experimental findings, the following conclusions can be drawn:

1. The mechanical performance of hydraulic lime-based mortars was strongly dependent on additive type and incorporation level. The reference mortar exhibited compressive and flexural strengths of 9.34 MPa and 2.36 MPa, respectively. Most modified mixtures developed lower mechanical strengths than the reference formulation, indicating that the incorporation of mechanically milled mineral additives or graphene nanoplatelets did not necessarily improve mechanical performance. The results demonstrated distinct, and frequently non-monotonic, relationships between additive dosage and strength development.
2. The incorporation of 3% injection mortar additive (IM-3%) provided the most favorable overall mechanical performance. This formulation achieved the highest mean compressive strength of 9.75 MPa, representing a 4.4% improvement relative to the reference mortar, together with a flexural strength of 2.97 MPa, corresponding to a 25.8% increase. In contrast, the lower IM dosages produced reduced compressive strengths, demonstrating a pronounced dependence on additive content. The favorable performance of IM-3% coincided with locally interconnected and comparatively compact morphological features observed through SEM. However, the available microstructural analyses did not establish a definitive mechanism responsible for the measured improvements.
3. Slaked construction lime and graphene nanoplatelets exhibited favorable flexural responses at the lowest investigated dosage. SCL-1% achieved the highest mean flexural strength of 3.02 MPa, corresponding to a 28.0% improvement, while its compressive strength increased by 2.7%. However, the relatively high coefficient of variation in flexural strength (29.3%) indicates substantial experimental variability and limits the certainty of this improvement. Similarly, GN-1% increased flexural strength by 19.1% but reduced compressive strength by 13.6%. Increasing the graphene nanoplatelet content beyond 1% did not maintain the flexural improvement, demonstrating that mechanical benefits were dependent on both dosage and loading condition. Although IM-3% and SCL-1% exhibited higher mean compressive strengths than the reference mortar, the corresponding increases of 4.4% and 2.7% represent descriptive differences rather than statistically confirmed improvements. Given the limited number of specimens (n = 3), additional replicate testing and inferential statistical analysis are required to verify the reliability of these differences.
4. The remaining mineral additives exhibited predominantly unfavorable mechanical responses. Mechanically milled hydraulic lime and repair mortar produced progressive reductions in compressive and flexural strengths as their incorporation levels increased. Brick powder exhibited gradual strength improvements within its dosage series; however, none of the BP-modified mixtures exceeded the corresponding reference strengths. Gypsum-based plaster-modified specimens disintegrated during water curing and were therefore excluded from mechanical testing. This behavior demonstrated inadequate stability of the investigated gypsum-containing formulations under the adopted curing conditions, although the underlying deterioration mechanism could not be conclusively identified.
5. Microstructural characterization revealed additive-dependent morphological and compositional differences without major changes in the principal crystalline characteristics of the hardened mortar matrices. SEM-EDS observations identified variations in particle arrangement, local clustering, and apparent matrix continuity. XRD patterns were predominantly characterized by carbonate-related reflections, while FT-IR spectra retained the principal carbonate- and silicate-associated absorption bands. The absence of distinct new crystalline phases or dominant spectroscopic features suggests that the observed mechanical differences cannot be explained solely by the formation of readily identifiable reaction products. However, the available analyses did not quantitatively establish the contributions of particle packing, porosity, hydration products, or additive-matrix interactions to mechanical performance.
6. The findings should be interpreted within the limitations of the experimental design. Although all mineral additives were subjected to controlled planetary ball milling, the absence of untreated additive counterparts and direct before-and-after powder characterization prevents the independent effects of milling from being established. The observed mechanical responses therefore reflect the combined influences of additive composition, dosage, and mixture proportioning rather than a separately quantified milling effect. Furthermore, the suitability of the investigated formulations for historic masonry conservation cannot be established solely from their mechanical and microstructural characteristics. The present experimental program was limited to additive contents of 1%, 2%, and 3%. The favorable flexural responses observed for SCL-1% and GN-1%, together with the non-monotonic strength trends of several formulations, indicate that the behavior at lower incorporation levels remains an important question. Future work will therefore investigate additive dosages below 1%, particularly for formulations that showed beneficial responses at the lowest tested content. Such investigations may help identify more suitable dosage ranges and clarify the relationship between additive content and mechanical performance, without presuming that further reductions will necessarily improve strength. Future investigations should include untreated and milled additive controls, particle-size distribution and specific surface area measurements, and complementary structural characterization. Additional studies addressing long-term mechanical performance, pore structure, durability, rheological behavior, moisture transport, and compatibility with historic masonry materials are also necessary before practical conservation applications can be recommended.

Overall, the findings demonstrate that the mechanical response of hydraulic lime-based mortars incorporating mechanically milled mineral additives and graphene nanoplatelets depends on additive composition, dosage, mixture proportions, and the characteristics of the hardened matrix. Among the investigated formulations, IM-3% emerged as the most promising candidate for further mechanical and microstructural investigation, while SCL-1% and GN-1% exhibited potentially beneficial, but more property-specific, responses. The study provides a comparative experimental basis for the development of modified hydraulic lime-based mortars and highlights the importance of balancing mechanical improvements with material compatibility and long-term performance requirements in historic masonry conservation.

## Figures and Tables

**Table 1:** Mixing ratios for the production of 1 m^3^ of mortar.

Constituent	Quantity	Unit
Hydraulic lime (HL)	1033	kg/m^3^
Water	362	kg/m^3^
Porcelain aggregate (Ø5 mm)	665	kg/m^3^
Water-to-hydraulic lime ratio (W/HL)	0.35	–
Additive content—1%	10.33	kg/m^3^
Additive content—2%	20.66	kg/m^3^
Additive content—3%	30.99	kg/m^3^

Note: Additive contents are expressed as percentages of the original hydraulic lime mass and represent additions rather than binder replacements. The calculated water-to-total-powder ratios are approximately 0.350, 0.347, 0.344, and 0.340 for PM and the 1%, 2%, and 3% additive mixtures, respectively.

**Table 2:** Compressive strengths of hydraulic lime-based mortar cube specimens at 28 days (MPa).

Compressive Strength (MPa)							
Additive Content (% by Mass of Reference Binder)	PM	HL	BP	RM	IM	SCL	GN
1%	9.34	7.91	7.07	7.78	7.25	9.59	8.07
2%		7.79	7.94	7.62	6.33	8.49	8.54
3%		7.73	8.50	7.44	9.75	9.29	8.22

**Table 3:** Flexural strengths of hydraulic lime-based mortar prism specimens at 28 days (MPa).

Flexural Strength (MPa)							
Additive Content (% by Mass of Reference Binder)	PM	HL	BP	RM	IM	SCL	GN
1%	2.36	1.50	1.03	1.33	1.76	3.02	2.81
2%		1.41	1.24	1.10	2.20	2.03	2.30
3%		0.97	1.33	1.00	2.97	2.11	2.14

**Table 4:** Mean compressive and flexural strengths, standard deviations, and coefficients of variation in hydraulic lime-based mortars at 28 days (n = 3).

		Flexural Strength (MPa)		Compressive Strength (MPa)	
Specimen Type	Additive Content (% *w*/*w* of Binder)	Mean ± SD	CoV (%)	Mean ± SD	CoV (%)
PM	-	2.36 ± 0.19	8.1	9.34 ± 0.83	8.87
HL	1%	1.50 ± 0.01	0.7	7.91 ± 0.19	2.38
	2%	1.41 ± 0.12	8.2	7.79 ± 0.65	8.36
	3%	0.97 ± 0.12	12.1	7.73 ± 0.97	12.59
BP	1%	1.03 ± 0.06	5.4	7.07 ± 0.84	11.83
	2%	1.24 ± 0.05	4.0	7.94 ± 0.47	5.92
	3%	1.33 ± 0.01	0.8	8.50 ± 0.33	3.90
RM	1%	1.33 ± 0.05	3.6	7.78 ± 0.15	1.97
	2%	1.10 ± 0.10	8.7	7.62 ± 0.52	6.88
	3%	1.00 ± 0.15	14.9	7.44 ± 0.51	6.86
IM	1%	1.76 ± 0.18	10.2	7.25 ± 0.29	4.00
	2%	2.20 ± 0.09	4.1	6.33 ± 0.67	10.63
	3%	2.97 ± 0.04	1.2	9.75 ± 0.19	1.95
SCL	1%	3.02 ± 0.89	29.3	9.59 ± 0.18	1.88
	2%	2.03 ± 0.24	11.7	8.49 ± 0.46	5.37
	3%	2.11 ± 0.23	10.9	9.29 ± 0.75	8.05
GN	1%	2.81 ± 0.05	1.8	8.07 ± 0.44	5.39
	2%	2.30 ± 0.09	3.9	8.54 ± 0.43	5.03
	3%	2.14 ± 0.18	8.2	8.22 ± 0.41	4.96

## Data Availability

The original contributions presented in this study are included in the article. Further inquiries can be directed to the corresponding author.
